# Recognition of polymorphic Csd proteins determines sex in the honeybee

**DOI:** 10.1126/sciadv.adg4239

**Published:** 2023-10-04

**Authors:** Marianne Otte, Oksana Netschitailo, Stefanie Weidtkamp-Peters, Claus A. M. Seidel, Martin Beye

**Affiliations:** ^1^ Institute of Evolutionary Genetics, Heinrich-Heine University, Düsseldorf, Germany.; ^2^ Center for Advanced Imaging, Heinrich-Heine University Duesseldorf, Germany.; ^3^ Institut für Physikalische Chemie, Heinrich-Heine University, Düsseldorf, Germany.

## Abstract

Sex in honeybees, *Apis mellifera*, is genetically determined by heterozygous versus homo/hemizygous genotypes involving numerous alleles at the single complementary sex determination locus. The molecular mechanism of sex determination is however unknown because there are more than 4950 known possible allele combinations, but only two sexes in the species. We show how protein variants expressed from complementary sex determiner (*csd*) gene determine sex. In females, the amino acid differences between Csd variants at the potential-specifying domain (PSD) direct the selection of a conserved coiled-coil domain for binding and protein complexation. This recognition mechanism activates Csd proteins and, thus, the female pathway. In males, the absence of polymorphisms establishes other binding elements at PSD for binding and complexation of identical Csd proteins. This second recognition mechanism inactivates Csd proteins and commits male development via default pathway. Our results demonstrate that the recognition of different versus identical variants of a single protein is a mechanism to determine sex.

## Teaser

Molecular recognition determines sex.

## INTRODUCTION

**Fig. 1.**
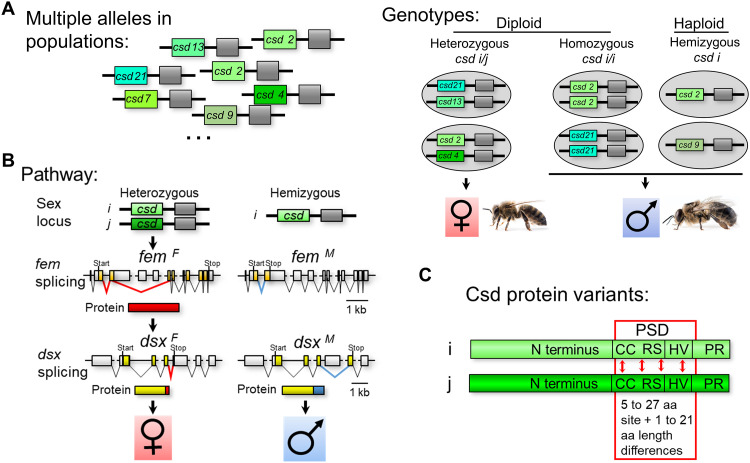
Complementary sex determination in the honeybee. (**A**) Multiple alleles of the complementary sex locus in the population. Heterozygosity and hemi/homozygosity determine sex. *i* and *j* symbolize different alleles. (**B**) The sex-determining pathway. The sex locus signal controls female-specific splicing of *fem* transcripts, which produce Fem proteins. Fem protein controls female development via the female splicing of downstream transcripts, including those of the *dsx* gene. Males result from default regulation. The genomic splicing scheme and proteins are shown. ^*F*^ and red color indicate female-specific regulated products; ^*M*^ and blue indicate male-specific ones. (**C**) Highly polymorphic Csd protein variants. The range of amino acid (aa) differences between Csd protein variants is presented. HV, hypervariable region domain; PR, proline-rich domain. *i* and *j*: different protein variants are shown by different green colors.

A primary decision process at the beginning of a sex-determining cascade controls the development of either females or males, which has important consequences for the anatomy, physiology, and behavior of the organism and has fascinated mankind since antiquity. A sex-regulating locus with multiple naturally occurring alleles (complementary sex determination) regulates sex in the honeybee (*Apis mellifera*; Fig. 1A) (*1*, *2*). Complementary sex determination promotes outbreeding and enables control over sex ratio under haplodiploidy (*3*–*7*). Males under haplodiploidy develop from unfertilized, haploid eggs representing only the female genome, while females develop from fertilized eggs (Fig. 1A). Complementary sex determination is at least common in hymenopteran insects, which represent approximately 150,000 species, including ecologically important bee, ant, and wasp species (*8*–*11*). Two different sex alleles at the sex locus (heterozygosity) determine femaleness. One allele (hemizygosity in haploids) or two identical alleles (homozygosity in diploids) determine maleness (Fig. 1A). However, the homozygous, diploid males carrying identical alleles do not survive or produce only diploid sperm (*10*, *12*), establishing a high cost for inbreeding. Typically, 11 to 19 sex alleles segregate in local breeding populations, which was functionally examined by crossing experiments and/or diploid male production in different local honeybee populations (*13*–*18*). At the species level, more than 100 sex alleles have been molecularly found (*19*, *20*), suggesting that at least 4950 allele combinations must exist. However, how 4950 known possible allele combinations determine only two sexes is not understood. The multiple alleles underlying this sex determination system suggest that the molecular regulation is distinct to those found in sex chromosome or sex-specific gene systems.

The complementary sex locus was mapped in the honeybee (*A. mellifera*) to a genomic region of approximately 50 kb, encoding at least three genes (*15*, *21*–*23*). One gene, the *c*o*mplementary sex determiner* (*csd*) gene, has highly polymorphic alleles, is consistently heterozygous in females and homozygous in diploid males, is essential for sex determination (Fig. 1, A and B), and thus fulfills the criteria for a gene involved in the process (*15*, *21*, *22*). The activity of the *csd* gene is required to determine femaleness, while its absence produces maleness (*15*, *22*). The *csd* alleles encode variants of an SR-type protein (*15*), which is involved in transcript splicing regulation. Amino acid differences between protein variants are mainly found in the RS (arginine/serine-rich) domain, while length variation is located in the HVR (hypervariable region) domain (Fig. 1C) (*16*, *17*). Together, the two domains essentially define the potential-specifying domain (PSD) because they harbor most of the polymorphism, are under strong balancing selection, and encode the specificity of an allele (*16*–*18*). Within the variable RS domain, a conserved CC (coiled-coil) motif is present (*22*).

The *csd* gene is required for the activation of the *feminizer* (*fem*) gene [an ortholog of the *transformer* (*tra*) gene] and *doublesex* (*dsx*) gene, which are key components of sex determination pathways (Fig. 1B) (*21*, *24*–*34*). The activity of *csd* mediates the female-specific splicing of *fem* transcripts (*fem^F^*), which encode Fem proteins (*21*, *35*). The Fem proteins direct the female-specific splicing of *dsx* transcripts (*dsx^F^*). In the absence of *csd* gene activity, the *fem* transcripts are spliced into the male form (*fem^M^*). *fem^M^* transcripts do not code for a protein, which results in the default regulation of *dsx^M^* transcripts. The sex-specific *dsx^M^* and *dsx^F^* transcripts encode Dsx proteins with sex-specific peptides at their C-terminal ends (*36*, *37*), which direct sex-specific reproductive organ development (*38*).

Despite progress in characterizing sex locus genes and alleles and the sexual regulation of downstream genes, molecular dissection of the complementary mechanism has previously been difficult. Here, we used transgenic methods, CRISPR-Cas9–mediated mutations (*38*, *39*) together with biochemical studies, to systematically dissect the molecular mechanism of complementary sex determination. To understand how complementary sex determination is regulated, we examined whether two different *csd* alleles are genetically essential and sufficient to activate the female pathway. We also characterized the molecular interactions between Csd proteins and their polymorphic sites and examined whether selective bindings regulate the activity of the protein. We found that complementary sex determination is based on a single gene, *csd*. The different and identical Csd proteins are distinctly recognized to regulate the activity of the protein in females and males. In females, the amino acid differences direct binding of different Csd protein variants via the conserved CC domain, which activates the protein. In males, the identical variants use other PSD elements for their binding, which inactivates the protein. These results show that the *csd* gene uses molecular recognition of different versus identical Csd proteins as a mechanism to control the sexual fate.

## RESULTS

### Two different *csd* coding sequences are essential and sufficient to activate the female pathway

Bees that are heterozygous at the sex locus develop into females, while bees hemi- or homozygous at the sex locus differentiate into males (Fig. 1A). At least three genes have been found in the complementary sex locus region. However, only the *csd* gene fulfills the criteria for a gene involved in the process (*15*, *21*). Most polymorphisms of the *csd* gene are found in the sequence coding for the RS and HVR domains, which, together, essentially define the PSD (*16*–*18*). Five– to 27–amino acid pairwise differences together with 1– and 21–amino acid pairwise length differences encoded by the PSD region define functionally distinct *csd* alleles in heterozygous sex locus genotypes (Fig. 1C). These alleles were defined by crossing studies using 13 alleles randomly sampled from a local population, which all determine female development in the heterozygous genotype (*18*). RNA interference (RNAi)–mediated knockdown of both *csd* alleles in females showed that the activity of the *csd* gene is required for female development, while it is dispensable for male differentiation (Fig. 1B) (*15*, *22*). However, these studies did not functionally address the question of whether, solely, the allele differences at the *csd* gene determine sex. This would require examining whether the combination of two different *csd* alleles is genetically essential and sufficient to determine femaleness.

**Fig. 2.**
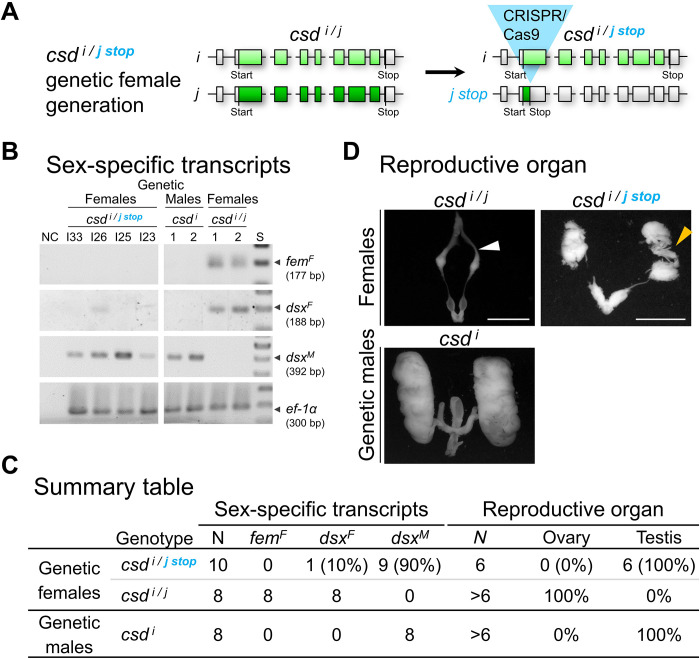
The combination of two different *csd* coding sequences is essential for female determination. (**A**) The generation of *csd ^i/j stop^* genetic (diploid) females. *i/j* and light/dark green color symbolize different alleles in the testing. Blue triangle, induced stop codon in exon 2 using CRISPR-Cas9. Polymorphisms in the *i* allele restricted mutations to the *j* allele as shown by deep sequencing of the target site using amplicons for each bee. (**B**) Sex-specific splicing of genetic female with the *csd ^i/j stop^* genotype at late larval/ early pupal stage. The mutations were independently induced for each bee. Resolved amplicons are presented as black/white negatives. Reverse transcription polymerase chain reactions (RT-PCRs) were semiquantitatively adjusted across individuals using *ef-1*α (elongation factor 1α) transcripts as a control. *fem^F^*, female *fem* transcript; *dsx^F^*/*dsx^M^*, female/male *dsx* transcripts; S, marker in 100–base pair (bp) steps; NC, negative PCR control. (**C**) The frequencies of female/male transcripts and reproductive organs in *csd ^i/j stop^* genetic females. (**D**) Reproductive organ of *csd ^i/j stop^* genetic females at the early pupal stage (reared on worker nutrition). Yellow arrowhead, horizontally packed testioles; white arrowhead, few vertically organized ovarioles. Scale bars, 2 mm.

To determine whether two different *csd* alleles are required to determine femaleness, we introduced early stop codons in the coding sequence of only one allele and this in heterozygous females (Material and Methods and Table 1). The sex-specific splicing of *fem* and *dsx* transcripts determines either female or male development (*21*, *22*, *38*). We thus examined whether the splicing shifted from the female to the male variant in the mutated genetic females as a measure for the loss of *csd* gene activity for female determination. We also studied whether male gonad development is a result. Stop codons in exon 2 were introduced in a single allele of the heterozygous genotypes using the CRISPR-Cas9 method and embryonic injections (*38*, *40*). This independently generated mutated female embryos, which were reared in the laboratory. We than identified those genetic females with an *i/j ^stop^ csd* genotype (Fig. 2A) by genotyping all treated females. Frequent polymorphism in exon 2 regularly limited mutations to a single allele. The heterozygous genotype and the stop codon in a single allele were confirmed using deep-sequenced amplicons of the target sites (fig. S1). In six individuals, the *j* allele was entirely mutated [no mosaicism (*38*)], while in four individuals, in addition to the stop mutation, WT (wild-type) sequences (the *i* sequences) were also found (fig. S1).

**Table 1.** Material and equipment. N/A, not applicable.

Reagent or resource	Source	Identifier
*Antibodies*		
Mouse monoclonal anti-myc antibody	Roche	Catalog no. 9E10
Goat anti-mouse antibody HRP conjugate	Sigma	Catalog no. 71045-M
*Bacterial strain*		
*E. coli* DH5α	Thermo Fisher Scientific	Catalog no. 18265017
Chemicals, peptides, and recombinant proteins		
Spodopan (proteinfreies Medium)	PAN-Biotech	Catalog no. P04-850100
Cas9	New England Biolabs	Catalog no. M0646
TRIzol	Thermo Fisher Scientific	Catalog no. 15596026
Heavy isotope–labeled synthetic peptide	Thermo Fisher Scientific	N/A
*Critical commercial assays*		
RiboMAX Kit	Promega	Catalog no. P1300
MEGAclear Kit	Thermo Fisher Scientific (Ambion)	Catalog no. AM1908
RevertAid First Strand cDNA Synthesis Kit	Thermo Fisher Scientific	Catalog no. 10680471
Eco RI	Thermo Fisher Scientific	Catalog no. ER0271
SaI I	Thermo Fisher Scientific	Catalog no. FD0644
T7-transcription/translation coupled reticulocyte lysate system	Promega	Catalog no. L4610
Amersham ECL Kit	Thermo Fisher Scientific	Catalog no. 12316992
peqGOLD Tissue DNA Mini Kit	VWR	Catalog no. 12-3496-02
DNA Polymerase I	Thermo Fisher Scientific	Catalog no. EP0041
Ribonuclease H	Thermo Fisher Scientific	Catalog no. 18021014
EZNA Cycle Pure kit	Omega Bio-Tek Inc., Norcross	Catalog no. D6492-01
*Experimental models: Cell lines*		
*Sf*21 cells	Invitrogen, Fischer Scientific	Catalog no. 10103722
*Oligonucleotide sequences 5′ to 3′ for sgRNAs and PCRs*		
Oligo_gRNA 2: GCATTAATTTGAATACCTTC	Eurofins	N/A
dsxM_f: CTATTGGAGCACAGTAGCAAACTTG; dsxM_r: GGCTACGTATGTTTAGGAGGACC	Eurofins	N/A
dsxF_f: CTATTGGAGCACAGTAGCAAACTTG; dsxF_r: GAAACAATTTTGTTCAAAATAGAATTCC	Eurofins	N/A
femM_f: TGAAGTTAATAACATATTTTTAATTCATCAATGAAG; femM_r: TGTACCATCTGAAGATTCTAATTTTTCG	Eurofins	N/A
femF_f: CTGATTTTTCAATATTTACAGCTAAAACTGTAC; femF_r: CAACATCTGATGAACTTAAACGG	Eurofins	N/A
*Recombinant DNA*		
pGBKT7 plasmid	TaKaRa	Catalog no. 630443
YFP (yellow fluorescent protein)	pBI121-35S H2B YFP	Provided by D. Schubert
Cerulean (fluorescent protein)	pCFP-C1 plasmid	Addgene; provided by S. Weidtkamp-Peters
PIZV5-His vector	Thermo Fisher Scientific (Life Technologies)	Catalog no. V800001
pFastBac-HTA vector	Thermo Fisher Scientific	Catalog no. 10584027
Baculovirus bacmid	Thermo Fisher Scientific (Life Technologies)	Catalog no. 10360014
*Software and algorithms*		
Multi Gauche	Fujifilm	Version 3.2
Systat	Systat	Version 13.1
IGV	Broad Institute	https://software.broadinstitute.org/software/igv/
Peak Scanner software	Thermo Fisher Scientific	Catalog no. 4381867
AnI; Margarita	Institute für Physikalische Chemie	www.mpc.hhu.de/software/mfd-fcs-and-mfis
Mascot 2.4 within Proteome Discoverer	Thermo Fisher Scientific, Germany	Version 1.4.1.14; www.thermofisher.com/order/catalog/product/OPTON-30810#/OPTON-30810
Against Swiss-Prot database (release 2018_07)	Expasy	www.expasy.org/resources/uniprotkb-swiss-prot
*Other*		
PVDF membrane (Merck Millipore)	Merck	Catalog no. IPVH00005
Lab-Tek Chamber Slides (Nunc)	Thermo Fisher Scientific	Catalog no. 154453
Rapid Separation liquid chromatography system	Thermo Fisher Scientific	www.thermofisher.com/de/de/home/industrial/chromatography/liquid-chromatography-lc/hplc-uhplc-systems/ultimate-3000-hplc-uhplc-systems/rapid-separation-rs-hplc-systems.html
Acclaim PepMap 100 C18 column (inner diameter, 75 μm; length, 25 cm; particle size, 2 mm)	Thermo Scientific	
Q-Exactive Plus mass spectrometer	Thermo Fisher Scientific	Catalog no. IQLAAEGAAPFALGMBDK
Olympus FV1000 confocal laser scanning microscope	Evident Olympus Deutschland GmbH, Wendenstr. 20, D-20097 Hamburg	www.olympus-lifescience.com/de/laser-scanning/
PicoQuant HydraHarp400 Multichannel Picosecond Event Timer & TCSPC Module	PicoQuant GmbH, Rudower Chaussee 29 (IGZ) 12489 Berlin, Germany	www.picoquant.com/products/category/tcspc-and-time-tagging-modules
PicoQuant Sepia PDL 828 Multichannel Picosecond Diode Laser Driver	PicoQuant GmbH, Rudower Chaussee 29 (IGZ) 12489 Berlin, Germany	www.picoquant.com/products/category/picosecond-pulsed-driver/pdl-828-sepia-ii-computer-controlled-multichannel-picosecond-diode-laser-driver
PicoQuant LDH Series Picosecond Laser Diode Head 440 nm for PDL 800-D / PDL 828	PicoQuant GmbH, Rudower Chaussee 29 (IGZ) 12489 Berlin, Germany	www.picoquant.com/products/category/picosecond-pulsed-sources/ldh-series-picosecond-pulsed-diode-laser-heads
PicoQuant PDM Series Single Photon Avalanche Diodes, Tau-SPAD	PicoQuant GmbH, Rudower Chaussee 29 (IGZ) 12489 Berlin, Germany	www.picoquant.com/products/category/photon-counting-detectors/pdm-series-single-photon-avalanche-diodes
Thorlabs PBS101–10 mm Polarizing Beamsplitter Cube, 420–680 nm	Thorlabs GmbH Münchner Weg 185232 Bergkirchen, Germany	www.thorlabs.de/thorproduct.cfm?partnumber=pbs101
Chroma 480/40x ET Bandpass	AHF analysentechnik AG Kohlplattenweg 18; DE-72074 Tübingen	www.ahf.de/produkte/spektralanalytik-photonik/optische-filter/einzelfilter/bandpass-filter/400-499-nm/2598/480/40x-et-bandpass?c=463
Illumina MiSeq	Roth	Catalog no. 2399
ABI 3130XL Genetic Analyzer	Applied Biosystems	Catalog no. 4352755
Segeberger nucs	Holtermann	Catalog no. 4935

Nine of 10 *csd ^i/j stop^* genetic females expressed the male *dsx^M^* transcripts and lacked the female *fem^F^* and *dsx^F^* transcripts, while WT females expressed only the female *fem^F^* and *dsx^F^* transcripts (Fig. 2, B and C). These numbers differed markedly and significantly (Fischer’s exact test, df = 1, *P* < 0.001). The *fem^M^* transcripts with their early stop codons were not informative and were excluded from the analysis because they were infrequently and only weakly detected in control males (fig. S2), suggesting more rapid decay of the male transcripts (*41*). The reproductive organs of *csd ^i/j stop^* females at larval or early pupal stage were of the male type and consisted of testes with multiple densely packed and folded testioles (*21*, *38*), while the *csd ^i/j^* controls had female reproductive organs (Fig. 2, D and C). This switch to male regulation and development due to the loss of function of a single allele in heterozygous females demonstrates that the combination of two different coding sequences is essential for female determination.

However, we were not able to rear these *csd ^i/j stop^* mutants to later pupal stage, suggesting reduced survival ship of mutants (chi-square test, df = 1, *P* < 0.01; table S1). Furthermore, their male reproductive organs were substantially smaller than those of WT males (Fig. 2D). This result suggests that the single-allele loss-of-function mutations also had, besides sexual, other effects that were not observed in the previous RNAi experiments, in which both *csd* alleles were knocked down (*21*, *22*).

**Fig. 3.**
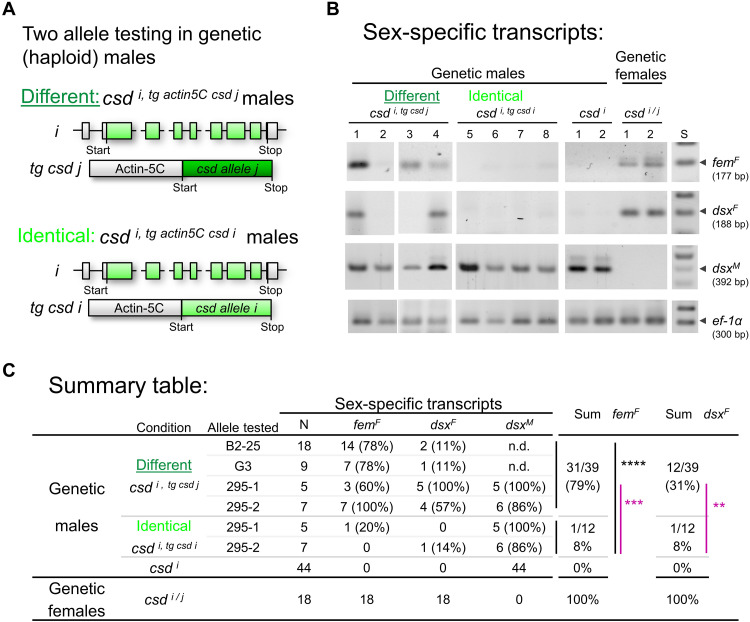
The combination of two different *csd* coding sequences is sufficient to produce female-determining activity. (**A**) Schematic of the transgenic experiments to examine the female-determining activity of two different versus two identical *csd* alleles in haploid males. Combinations of endogenous allele and transgenic allele were tested. *i/j* and light/dark green color symbolize coding sequences from different alleles; tg, transgene; long gray box, actin-5c promoter. (**B**) Female- and male-specific splicing of the *fem* and *dsx* transcripts in the transgenic males at larval stage. Size-resolved amplicons from RT-PCRs, which were semiquantitatively adjusted across individuals using *ef-1*α transcripts as a control. The numbers above the lanes indicate RT-PCRs from single bees. The pictures are black/white negatives. *^F^*, female; *^M^*, male transcript. (**C**) Summary table of the allele combinations examined. The feminizing activity in transgenic males with two different (*csd ^i, tg actin5C csd j^*) or two identical (*csd ^i, tg actin5C csd i^*) *csd* coding sequences. Males (*csd ^i^*) and females (*csd ^i/j^*) were WT controls. Black, ****P < 0.0001; purple, comparison of the identical alleles tested under both different and identical allele combinations: **P < 0.01 and ***P < 0.001.

To determine whether two different *csd* alleles are genetically sufficient to provide female-determining activity, we transgenically expressed a second naturally occurring *csd* allele in haploid males under the control of the honeybee promoter actin5C (Fig. 3A, Material and Methods, and Table 1) (*39*). We than studied whether these transgenic males show a shift from male to a female splicing pattern of the *fem* and *dsx* gene in the larvae as a direct measure of Csd protein activity for sex determination (*21*, *22*, *38*). To do so, we generated genetically transformed queens, which we treated with CO_2_ to obtain solely haploid male progeny (*39*). Because the queens were transgenic mosaics, only approximately 10% of the few male progenies carried the transgene as revealed by genotyping (*42*). To mimic the various heterozygous *csd* genotypes, we transgenically tested four different *csd* alleles in combination with various endogenous alleles (*csd ^i, tg actin 5C csd j^*; figs. S3 and S4). This effort resulted in 39 transgenic genetic males. To mimic the smaller number of homozygous genotypes, we transgenically tested two alleles that were identical with the endogenous allele (*csd ^i,tg actin5C csd i^*; figs. S3 and S4). The latter experiment required preselection of alleles and crossing experiments, which resulted in 12 *csd ^i, tg actin5C csd i^* genetic males with identical allele combinations. These overall low numbers of transgenic males make additional examinations of sexual development in adults impossible because the laboratory rearing generates a loss of 80 to 90% of the bees.

We found that the combinations of two different alleles resulted in marked feminization of males. Thirty-one of 39 (79%) of the *csd ^i, tg actin5C csd j^* genetic males had female-specific *fem^F^* (Fig. 3, B and C). This proportion markedly and significantly differed from the result for the control, with only 1 of 12 (8%) *csd ^i,tg actin5C csd i^* genetic males expressing *fem^F^* (Fisher’s exact test, df = 1, *P <* 10^−4^; Fig. 3, B and C). Those alleles, which we examined in both, different, and identical combinations (alleles 295-1 and 295-2), showed the same pattern [Fisher’s exact test, df = 1; *Fem^F^*: 77% versus 8% (*P <* 0.001); *Dsx^F^*: 69% versus 8% (*P <* 0.01); Fig. 3C]. These results suggest that two *csd* coding sequences were sufficient to substantially feminize genetic males. We conclude that, solely, the amino acid differences between two Csd protein variants regulate the activity that directs female splicing. However, the shift to female splicing and, thus, feminization in males were not complete, possibly because we cannot fully mimic the heterozygous genotype of diploids in our transgenic testing with the actin5c promoter in haploids.

Collectively, these results show that the combination of two different *csd* coding sequences are necessary and sufficient for the feminization at the level of *fem* splicing, the key decision process for sexual development, while the combinations of identical or single sequences are not. Because the coding sequence is required, this result suggests that the combination of different versus identical Csd protein variants regulate the activity of Csd proteins and the female pathway. We conclude that a mechanism that discriminates different versus identical protein variants is used to determine sex.

### Different and identical Csd protein variants form trimeric complexes

**Fig. 4.**
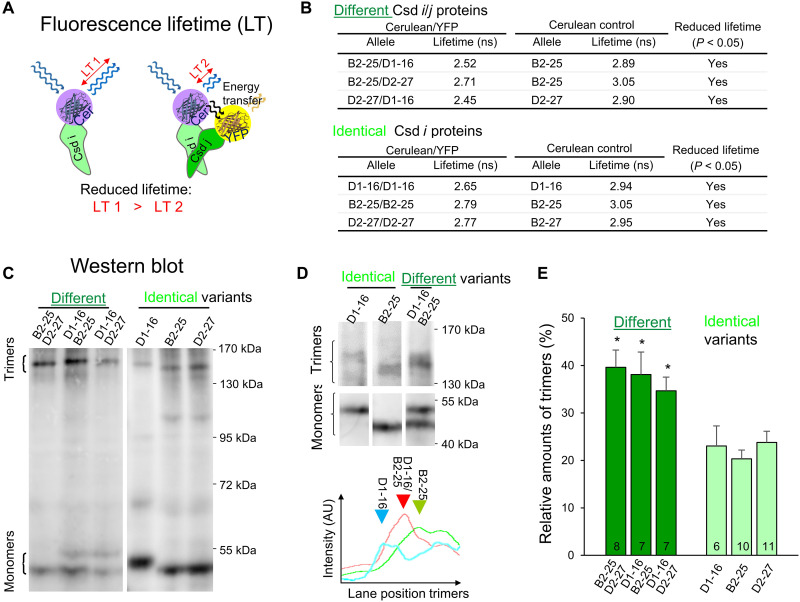
Trimeric complex formation between different and identical Csd protein variants. (**A**) Schematic of the fluorescence lifetime (LT) reduction upon protein binding via FRET. LT was measured using fluorescence lifetime imaging microscopy (FLIM). (**B**) LT values of the Csd protein variants that were fused with either cerulean protein or YFP. Proteins were expressed in *Sf21* insect cells using a baculovirus system. Values from *n* ≥ 6 per condition were compared using a *t* test. (**C**) Example of a Western blot using oligomer-forming conditions. Three different protein variant combinations and three identical Csd protein variants were expressed. Myc-tagged Csd proteins were resolved in 8% acrylamide gels and stained with anti-myc antibody. The complexes equal in size to trimers and monomers are marked. (**D**) Highly resolved trimeric complexes of different and single-protein variant expression using 6% acrylamide gels. All lanes are from the same Western blot. Bottom: Detected intensities along the position in each lane (lane profile) of the Western blot shown in (D). Arrows indicate the peak intensities. AU, arbitrary units. RU, relative units. (**E**) The relative amounts of the detected trimeric complexes. The number of replicates is indicated. The SEM is displayed. *z* test, *P < 0.05.

We propose that selective binding between different and between identical Csd protein variants allows specific recognition, which is used to determine sex. Protein binding studies turned out to be difficult because protein expression from plasmid-based vectors in prokaryotic and eukaryotic cells failed. However, persistent efforts using baculovirus- and in vitro–based systems allowed binding studies in cells and in vitro with weakly expressed Csd proteins (Material and Methods and Table 1). To examine whether different Csd protein variants selectively bind and form only heteromeric protein complexes, we studied the lifetime of fluorescence signals (Material and Methods and Table 1). Three Csd protein variants derived from different branches of the allele genealogy (*16*, *17*) were fused with either yellow fluorescent protein (YFP) or cerulean fluorescent protein and coexpressed from a single vector in insect cells. Upon their interaction in the cells, energy is transferred during fluorescence imaging microcopy, which reduces the lifetime of the fluorescence signal because of Förster resonance energy transfer (FRET; Fig. 4A) (*43*). We found that the lifetime of the fluorescence signal was significantly reduced for combinations of both different and identical protein variants (t test, *P* < 0.05; Fig. 4B and fig. S4) (*44*). This result showed that heteromeric and homomeric Csd protein complexes were formed, suggesting that the formation of heteromeric instead of homomeric complexes is not a mechanism for recognition. An alternative, possible recognition mechanism is that the number of proteins involved in complexation differs in heteromers and homomers. To determine whether the number of Csd proteins in heteromeric complexes and that of homomeric complexes differ, we expressed Csd proteins in vitro and studied Csd complexes using oligomer-forming conditions. We used reticulocyte lysates for the in vitro translations, which have the ability to maintain sulfhydryl groups in proteins in a reduced state. We found complex formation conditions, if we used very low denaturation conditions in the SDS–polyacrylamide gel electrophoresis (SDS-PAGE), native loading buffers (*45*), and detection using Western blots (material and methods, Table 1). For the combinations of different Csd protein variants and for single–Csd protein variants, we detected trimer-sized complexes (Fig. 4C), indicating that the number of Csd proteins involved is not a mechanism for specific recognition. To confirm that the different Csd protein variants form trimeric complexes, we examined Csd proteins with substantial length differences at high resolution. We observed that the peak intensities for the trimeric complexes from different protein expressions were of intermediate sizes compared to those from single-protein variants (Fig. 4D and fig. S5), suggesting that heterotrimeric complexes were formed. We also measured the relative amounts of trimers that were detected. We observed that these values were higher in combinations of different variants than in single-variant ones (*z* test, z > −3.4, *P* < 0.05; Fig. 4E), suggesting that the nature of the binding between different and identical Csd proteins differs. Collectively, the in-cell and in vitro binding studies demonstrate that Csd proteins are binding to each other. However, different versus identical protein variants are not discriminated via distinct complexations.

The *csd* gene evolved via gene duplication from an ancestral copy of the *fem* gene (*22*, *46*), providing an opportunity to study the evolutionary origin of trimeric complexation. To understand whether the trimeric complexation of Csd proteins is a novel evolved feature, we examined the complexation of Fem proteins. We found that Fem proteins formed low amounts of trimeric complexes (fig. S6), suggesting that the common ancestor protein of Csd and Fem already formed trimeric complexes.

### Polymorphisms regulate selective binding at PSD

**Fig. 5.**
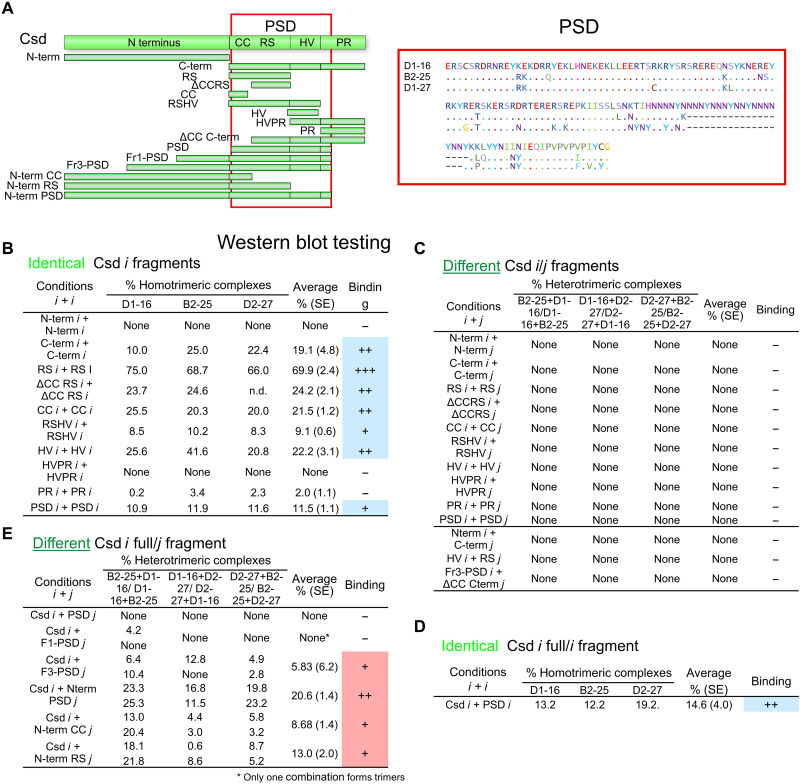
The presence or absence of polymorphisms controls nonbinding and binding at the PSD. (**A**) Schematic of the fragments used in the Western blot experiments. The amino acid polymorphisms at PSD of the protein variants used are shown. (**B** to **E**) The relative abundance of trimeric complexes using oligomer-forming conditions in SDS-PAGE. Proteins were detected in Western blots. Three different protein variant combinations and three identical variants were examined. The SEM with *n* ≥ 6 is shown. (B) Homotrimers are formed between elements of PSD. (C) Heterotrimers are not formed between elements of PSD. (D) PSDs form homotrimers together with full-length proteins. (E) The N-terminal/CC domain is a minimal segment for heterotrimer formation together with full-length proteins. The two values shown in (D and E) represent the measures of testing the two possible variant combinations of full-length proteins with fragments.

We next proposed that the sites for different and identical Csd variant bindings may differ, which establish a mechanism for recognition. Thus, we next examined the binding abilities of distinct domains of the Csd proteins. We quantified the amount of hetero- and homotrimeric complexes these domains formed in the Western blots under the oligomer-forming (*45*) condition (Fig. 5A, fig. S4, and table S2). PSDs from single–Csd protein variants formed substantial amounts of homotrimers (11.5% relative amount; Fig. 5B). Within the PSD, the RS domain showed the largest amount of trimer formation (69.9%), followed by ΔCC-RS (24.2%), the HVR (22.2%) domain, and the domain with the CC motif (21.5% relative amount; Fig. 5B and fig. S7). In addition to the PSD, the other domains, namely, the PR and the N-terminal domain, formed no homomeric complexes. For the combinations of different protein variants, we found that the PSDs formed no heteromeric complexes (Fig. 5C and fig. S8). However, the two PSD variants formed homotrimeric complexes according to their size difference (fig. S8). This lack of binding between different variants was also true for elements of PSD (RS, ΔCC-RS, CC, and HVR) and the N-terminal and PR domains (Fig. 5C). Next, we found that nonhomologous domain combinations also did not form heteromeric complexes because we observed no binding between N-terminal and C-terminal fragments, HVR and RS domains, and other nonhomologous fragments from different variants (Fig. 5C). To determine whether PSD polymorphism-dependent binding is a shared feature among alleles, we examined the binding of six other PSD variants in various combinations (*18*). We also found that these other PSD variants consistently formed homotrimeric but not heterotrimeric complexes (table S3). Collectively, these results suggest that the amino acid polymorphism at PSD encode a nonbinding ability between different variants. In the absence of these polymorphisms between identical variants, the PSD and its elements encode binding ability.

Because the PSD with its polymorphic differences did not provide a binding ability between different variants, we proposed that heteromeric binding must be achieved via another domain, which is conserved and shared among Csd protein variants. Bioinformatic predictions identified a conserved CC binding motif within PSD (*22*), which is shared among the variants. This CC motif evolved after the *csd* gene originated by gene duplication (*22*, *46*). The CC motif can provide a shared binding ability because the CC domain forms α-helical structures with hydrophobic surfaces. Possibly, this binding domain is specifically used if the polymorphic differences at PSD prevent binding, suggesting that different segments are required for heteromeric binding. To identify such heteromeric binding, we identified the minimal segment that is essential for the binding of two Csd protein variants. This required binding studies that compare the binding between full-length protein and fragments. We first showed that in this changed experimental setting, the PSD and full-length proteins formed only homotrimeric but not heterotrimeric complexes (Fig. 5, D and E), confirming our previous PSD binding results. Next, we added the N-terminal fragment to the PSD and observed heterotrimer formation (Fig. 5E). Then, we added only the CC motif to the N-terminal fragment and found that the N-CC fragment and full-length proteins formed heterotrimers (8.7% relative amount), suggesting that at least one element in the N-CC sequence can provide heterotrimeric binding ability (Fig. 5E and fig. S9). Next, we asked whether adding the most polymorphic elements of PSD (ΔCC-RS and HVR) can enhance this heteromeric binding. Adding these polymorphic elements resulted in an increase in heterotrimer formation (Fig. 5E), suggesting that the polymorphic elements with no heteromeric binding abilities enhance the formation of heteromeric complexes.

Collectively, these results indicate that identical Csd variants with identical amino acid residues selectively bind at PSD. In the case of different Csd variants, the two polymorphic PSD variants do not bind. However, polymorphic differences enhance selective binding to another domain located in the N-CC fragment. Because the only conserved and predicted binding motif in the minimal N-CC fragment was the CC motif, we next investigated the binding function of this CC motif.

### The CC domain is the shared binding element for the selective binding of different Csd protein variants

**Fig. 6.**
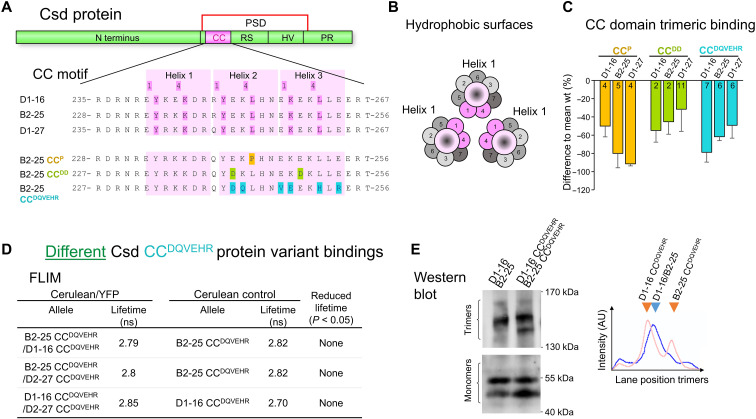
The CC motif encodes the shared heterotrimeric binding ability and is essential for the binding of different Csd protein variants. (**A**) The position and sequences of the CC motifs in the different Csd protein variants. Light pink, heptad repeats; dark pink, hydrophobic/amphipathic amino acid residues at positions one and four. Further below, the examined amino acid replacements are shown for only one allele. (**B**) Schematic presentation of the proposed trimeric binding. Usually, heptad repeats form α helices with hydrophobic surfaces (displayed in dark pink). Helices from different Csd proteins (shown for helix 1 only) interact to form a trimeric complex (the CC structure). (**C**) The quantitative difference in homotrimer formations of mutated versus WT sequences using Western blots from SDS-PAGE using oligomer-forming (low denaturation) conditions. The SEM and the number of replicates are indicated. (**D**) Fluorescence lifetime values derived from combining two different Csd-CC^DQVEHR^ protein variants. Values from *n* ≥ 6 cells per condition were compared using a *t* test. (**E**) To the left: Highly resolved trimeric complexes (6% acrylamide gels) derived from combining two Csd-CC^DQVEHR^ or two WT Csd protein variant expressions. Lanes are from the same Western blot. Western blots were from SDS-PAGE using oligomer-forming conditions. To the right: Detected intensities along the position in each lane (lane profile) of the Western shown to the left. Arrows indicate the peak intensities.

Bioinformatic studies predicted that each of the three heptad repeats from the CC motif can form α helices (Fig. 6, A and B). The amino acid residues at positions one and four of each repeat are hydrophobic or amphipathic, which establish hydrophobic binding surfaces. The other positions are hydrophilic/amphipathic. These α helices can wrap around each other along hydrophobic surfaces (Fig. 6B) to build trimeric complexes (a CC structure) with hydrophilic residues at the outer surface (*47*). To determine whether the CC motif has the capacity to control trimeric binding, we replaced amino acids at core positions and studied the formation of homotrimeric complexes of the CC fragment using Western blot analysis under the oligomer-forming conditions (*45*). Replacing lysine (L) with proline (P), which disrupts the helical structure (*48*) in the second heptad repeat (CC^P^), substantially reduced trimerization by 30 to 55% for three variants compared to the WT (Fig. 6C). Furthermore, the replacement of glutamic acid (E) with the shorter aspartic acid (D) (CC^DD^) at two core hydrophilic positions reduced trimer formation by 50 to 90%. Next, we replaced the six amino acid residues that evolved with the origin of the *csd* gene with those found in the paralog Fem protein (CC^DQVEHR^). This changed two hydrophobic/amphipathic residues to hydrophilic residues and one hydrophilic residue to a hydrophobic residue. These replacements resulted in a reduction of 50 to 80% in trimer formation (Fig. 6C). Collectively, this reduction in the response to compromised CC core positions suggests that the CC motif establishes a trimeric binding ability, which is shared among the different Csd protein variants.

Last, to determine whether this conserved CC motif enables the binding of different Csd protein variants, we examined whether the motif is essential for the heteromeric complexation. We functionally compromised the CC motif using DQVEHR replacements and studied the heteromeric formation of full-length proteins. We found that the fluorescence lifetimes of different Csd-CC^DQVEHR^ protein variant combinations in insect cells were not reduced (Fig. 6D), while the fluorescence lifetimes of different variants with the WT sequence were reduced (*P* < 0.05; Fig. 4B). This result showed that the CC motif is essential for the heteromeric complexation of different Csd protein variants. Next, we examined the sizes of the complexes from CC mutated variants using Western blots and oligomer-forming conditions (*45*). We found that two different Csd-CC^DQVEHR^ variants formed two trimeric complexes of distinct sizes, while the WT variants formed a complex of intermediate size (Fig. 6E). This result suggests that the mutated proteins formed no heteromeric complexes, which would have an intermediate size. Instead, they form two separate homomeric complexes of distinct sizes. To examine whether the two complexes are homotrimeric ones, we examined the oligomerization of single Csd-CC^DQVEHR^ protein variants. We found that single Csd-CC^DQVEHR^ protein variants formed trimeric complexes (fig. S10). These results suggest that the two complexes of Fig. 6E are homomeric complexes and that the CC motif is not required for the homotrimerization. Collectively, these binding results from fluorescence lifetime imaging microscopy and Western blot analyses indicate that the CC domain is specifically essential for the binding of different Csd protein variants. The specificity of the mutational change that results in loss of complexation further suggests that the hydrophobic surfaces and not covalent binding (e.g., disulfide bounds) is used for the heteromeric binding. The CC motif is dispensable for homomeric complexation, which requires other binding elements in the PSD. We conclude that the polymorphic differences direct a selective CC domain binding for the heteromeric complexation. This selective CC binding establishes a mechanism that can specifically recognize different Csd protein variants.

### The selective CC domain binding of Csd protein variants is essential for female determination

**Fig. 7.**
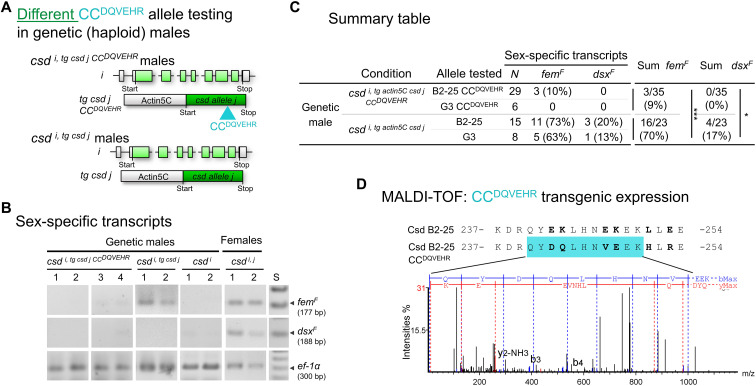
The CC domain binding of different protein variants is essential for female determination. (**A**) Schematic of the transgenic experiments to examine the function of the CC domain for the female-determining activity using *CC^DQVEHR^* replacements. Combinations of endogenous allele and transgenic allele were tested in haploid genetic males. *i/j* and light/dark green color symbolize the different *csd* coding sequences in transgenic males. Blue arrowhead, the introduced replacements compromising the CC domain. long gray box, actin-5c promoter. (**B**) Female- and male-specific splicing of the *fem* and *dsx* transcripts in the transgenic males at larval stage. Resolved amplicons as black/white negatives. RT-PCRs were semiquantitatively adjusted across individuals using *ef-1*α (*elongation factor 1α*) transcripts as a control. (**C**) Summary table of the allele combinations tested. The feminizing activity in transgenic genetic males with two different coding sequence and compromised CC domain (*csd* ^*i*, *tg csd j CC DQVEHR*^) and with two different WT alleles (*csd ^i, tg actin5C csd j^*). Males (*csd ^i^*) and females (*csd ^i/j^*) were WT controls. (**D**) Detection of CC^DQVEHR^ peptide in *csd* ^*i*, *tg csd j CC DQVEHR*^ males. MALDI-TOF fragment spectra of the peptide (blue box), which matches the spectra of a synthetic, identical, but isotope labeled peptide (fig. S12).

To understand whether this selective CC domain binding is the specific recognition mechanism for Csd protein activation and female determination, we transgenically expressed another Csd protein with a mutated CC motif in haploid genetic males. We generate transgenic queens, which carried another *csd* sequence variant and CC^DQVEHR^ coding changes (Fig. 7A). We than studied the transcript splicing of the *fem* and *dsx* genes in the transgenic haploid genetic male progeny (*csd ^i, tg actin5C csd j CC DQVEHR^*) as a direct measure of Csd protein activity. If the CC domain binding is necessary for the female-determining activity of different Csd proteins, then we expect to see a shift to male-specific splicing, despite the expression of the two Csd variants. This experiment thus aims to examine whether the use of the CC domain binding is the recognition mechanism, which is used to activate different Csd protein variants.

We found that the CC domain mutations substantially and significantly reduced female-specific splicing in *csd ^i, tg actin5C csd j CC DQVEHR^* genetic males compared to the control of transgenic genetic males (*csd ^i, tg actin5C csd j^* males) with different WT *csd* alleles and functional CC domains (Fig. 7, B and C, and fig. S11). Only 9% of the transgenic genetic males with the compromised CC domain showed female *fem* transcript splicing (*fem^F^*) compared to 70% of the control transgenic genetic males with the functional CC domain (Fisher’s exact test, df = 1, *P* < 10^−5^). For *dsx* transcripts, the values also differed (Fisher’s exact test, df = 1, *P* < 0.03). These results indicate that feminization activity was substantially lost because of the malfunction of the CC domain. Next, we determined whether this loss of activity due to the malfunctioning CC domain was similar to the one observed if two identical Csd proteins were expressed (the *csd ^i, tg actin5C csd i^* genetic males). This comparison will show whether the loss of activity due to the malfunctioning CC domain was similar to the level of background activity, which we observed in transgenic testing of identical alleles. We found that the female-specific splicing in *csd ^i, tg actin5C csd j CC DQVEHR^* genetic males was not different from genetic males expressing two identical proteins (*csd ^i, tg actin5C csd i^* males from Fig. 3C; Fisher’s exact test, df = 1, *P* > 0.8). This result indicates that the female-determining activity, which was gained through expressing different versus identical Csd variants, was entirely lost because of the malfunctioning of the CC domain. Because this loss of activity could also result from a lack of *csd j CC^DQVEHR^* allele expression, we next confirmed that the Csd CC^DQVEHR^ proteins were expressed using matrix-assisted laser desorption/ionization–time-of-flight (MALDI-TOF) fragment spectrum analysis. We detected the fragment spectra of the peptide carrying the amino acid replacements (Fig. 7D and fig. S12), indicating that the Csd CC^DQVEHR^ proteins were expressed. Together, these results suggest that selective CC domain binding of different variants is essential to activate Csd proteins and to determine femaleness. Because the CC domain binding is dispensable for the identical protein variant binding and activation, this suggests that other homomeric binding elements of PSD (RS and HVR domain; Fig. 5B) ensure homomeric complexation, which results in the inactivation of Csd proteins and the male determination by default. We conclude that different and identical Csd protein variants are recognized by distinct binding mechanisms, which control the sexual fate.

## DISCUSSION

### Recognition of different versus identical Csd protein variants determines activity and sex

In 1845, 60 years before the discovery of sex chromosomes (*49*), Dzierzon discovered that fertilization in honeybees can determine sex (*5*), which led to the discovery of haplodiploid reproduction (*3*, *4*, *6*, *7*), complementary sex determination (*1*, *2*, *8*–*11*), and the *csd* gene (*15*, *21*, *22*). However, despite a long history of research, the mechanism by which more than 4950 known possible allele combinations determine only two sexes has been unknown. In this study, we characterized its molecular control.

In this study, we mutated single *csd* alleles in the heterozygous females and found a shift to male regulation and male development. We transgenically expressed another *csd* allele in haploid genetic males and found a shift to female regulation. This genetic essentiality and sufficiency studies in honeybees suggest that complementary sex determination is based on the combination of alleles of a single gene, *csd*. The transgenic work also showed that the shift to female splicing in transgenic haploids was not complete [the splice regulation in female diploids involves regulatory feedback at the level of *fem* transcript splicing (*21*)], indicating that we cannot fully mimic the diploid female condition using transgenes in haploid genetic males. Possibly, the haploid bees lack factors that are only expressed in diploids as our embryonic gene expression study suggests (*50*). However, the genetic work in this study suggests that the gene products expressed from different and identical *csd* allele combinations must be recognized to determine sex.

**Fig. 8.**
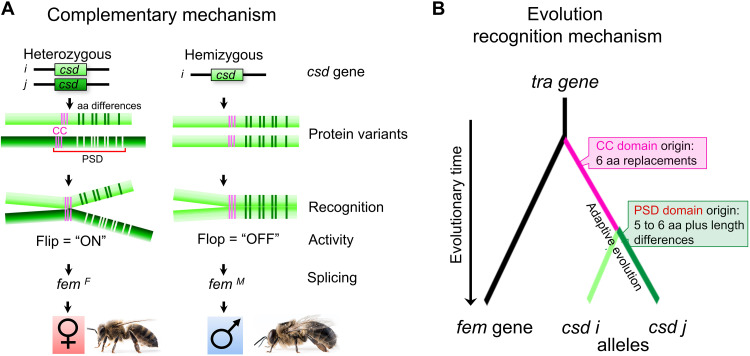
The molecular regulation and evolution of complementary sex determination. (**A**) The molecular basis of complementary sex determination in the honeybee. The sex locus expresses either different or identical Csd protein variants, which determine the sex. The different protein variants in females and identical variants in males are recognized by distinct bindings. In females, the amino acid differences at PSD of different Csd variants triggers the selection of CC domain binding for heteromeric complexation. In males, the absence of the amino acid differences of identical variants mediates binding at other PSD elements for homomeric Csd protein complexation. These distinct recognitions produce two possible binding states of Csd proteins (flip/flop mechanism of binding) that switch the activity state of Csd proteins either ON or OFF. The active Csd proteins (ON state) direct the female-specific splicing of the *fem* gene transcripts, a component of the conserved sex determination pathway. The inactive Csd proteins (OFF state) ensure male-specific splicing, which results by default regulation. *i/j* different *csd* alleles. Light versus dark green colors, different *csd* alleles/Csd proteins. (**B**) The molecular evolution of complementary sex determination. Two key components of the recognition mechanism newly and adaptively evolved after the *csd* gene evolutionary originated by gene duplication from the *fem*/*tra* progenitor gene. The evolution of the CC domain by amino acid replacements established a shared binding site among the Csd protein variants. The amino acid and sequence length difference establish between diverging PSDs a nonbinding ability, which is used to direct selective CC domain binding. The amino acid (aa) values are the changes for the formation of the CC domain (*22*) and the adaptive changes for the functional divergence of PSDs (*18*).

The results from the binding and functional studies than showed that specific recognitions of different versus identical Csd protein variants are used to determine sex. The different Csd protein variants (expressed from heterozygous complementary sex locus) are recognized by CC domain binding that activates the Csd proteins and, thus, the female pathway (Fig. 8A). In this case, the amino acid differences between PSDs determine nonbinding ability, which directs selective CC domain binding. Conversely, the identical protein variants (expressed from hemi- or homozygous locus) are recognized by other PSD bindings that we demonstrated by RS and the HVR domain homomeric binding (Fig. 5B). This other binding requires identical amino acid residues to inactivate the Csd proteins that ensure the default regulation of the male pathway (Fig. 8A). This flip-or-flop mode of Csd protein bindings establishes the recognition mechanisms that discriminate whether either different or identical protein variants are expressed. We then found that these recognitions regulate the “ON” or “OFF” female-determining activity of Csd proteins (Fig. 8A). In the ON state, Csd proteins direct the female splicing of *fem* transcripts. In the OFF state, the Csd proteins ensure that the *fem* transcripts are spliced into the male variant by default regulation. This *fem* transcript splicing is thereby the key process, which translate Csd’s ON or OFF activity into sex-specific gene regulation and the sexual development (Figs. 1B and 8A) (*21*, *22*). We propose that the distinct bindings form conformational differences between hetero- and homomeric complexes, which determine whether Csd protein activity is ON or OFF.

It was until now unknown how more than 4950 possible *csd* allele combinations can determine two sexes. Such a mechanism must specifically discriminate more than 4950 different versus 100 identical protein variant combinations to regulate Csd activity. The recognition mechanism that we found provides understanding how the enormous surplus of different versus identical protein variant combinations can firmly be discriminated and how this regulates protein activity. We found that the polymorphisms do not specify distinct binding states for each combination of different variants. Instead, amino acid differences direct the selection of the conserved CC domain binding. This CC domain binding establishes one shared binding and active state and among the enormous number of variant combinations (Fig. 8A). That the polymorphic amino acid residues of Csd proteins do not form matched binding abilities make this molecular recognition mechanism novel compared to other allele-based recognition mechanisms. Other molecular recognition systems are used to regulate the antigen representation in vertebrates or the self-incompatibility in plants and basidiomycetes (*51*–*57*). However, these other recognition systems (i) use matched binding between the polymorphic residues of proteins, and (ii) they require binding between two or more polymorphic proteins. To the contrary, the Csd recognition system (i) uses the nonbinding abilities of the polymorphic residues for specification of other domain bindings, and (ii) it uses variants of only single protein.

The *csd* alleles are maintained in the population over expanded evolutionary time (*17*). Rare *csd* alleles in the population produce fewer diploid males than frequent ones. Thus, they have an evolutionary advantage and increase over time in frequency. This balancing mode of selection maintains *csd* alleles over expanded periods of time in the population despite the counteracting force of genetic drift (*17*). However, under this selection regime as well, new *csd* alleles can mutationally originate and thus form two separate *csd* alleles from a common ancestor allele. Other *csd* alleles might be lost because of genetic drift. Evolutionary sequence analysis of *csd* alleles showed that the average time period that is required to split alleles into separate ones (coalescence time) was unexpectedly short compared to other loci under strong balancing selection, which suggest a high turnover rate of *csd* alleles in the population (*17*). This was most prominently observed by the absence of trans-specific alleles (the alleles are not found in other *Apis* species) (*17*). Previous modeling work showed that besides the small population sizes in honeybees, a higher mutational origin rate also would substantially reduce the coalescence time and would increase the turnover rates (*17*). The recognition mechanism of Csd proteins now provides molecular understanding that new *csd* alleles can originate at a high rate. Studies on the mutational divergence of protein domains showed that mutations favor the formation of nonbinding domains over binding domains (*58*). The favored mutational outcome suggests that a mechanism that uses nonbinding domains for the recognition of polymorphic protein differences would have a higher mutational origin rate for new alleles than those that use matched bindings between the polymorphic residues. In the case of Csd protein recognitions, we see that the polymorphic protein differences encode nonbinding domains, which are used to direct selective CC domain binding for recognition. Thus, the recognition of Csd proteins is a mechanism that specifically favors the mutational origin of new alleles.

Collectively, our results are a further demonstration that binding domains are important elements in molecular recognition. However, in the example of Csd proteins, we see that nonbinding domains are functionally relevant and have unappreciated features in the recognition process.

### Molecular recognition of proteins is a novel mechanism to determine sex

Progress has been made in identifying the molecular underpinnings of sex determination in vertebrates and invertebrates. One common principle that emerged from these studies is that sex-specific gene transcription is a mechanism to determine whether female and male development is executed (*59*–*64*). This sex-specific transcription of a gene is usually established through its sex-specific inheritance, which is realized through its location on sex- or neosex-specific chromosome (such as Y chromosome in males or W chromosome in females) (*28*–*30*, *59*–*63*, *65*–*71*). The sex-specific inheritance limits gene transcription and activity to only one sex and not the other, which controls the decision of either female or male development.

With the *csd* gene, we characterized a previously unknownmechanism in which molecular recognition of proteins determines sex (Fig. 8A). No sex-specifically inherited and expressed gene or alleles are used. Instead, the decision is made on the posttranslational level by recognizing different versus identical Csd protein variants. Despite this alternative principle of sex determination, the Csd mechanism still regulates an evolutionary shared downstream pathway consisting of the *fem/tra* gene and the *dsx* gene in invertebrates with its orthologous DM domain gene in vertebrates (*21*, *24*–*34*, *72*). *dsx*/DM domain genes are integral to sexual development in many metazoans, and they coordinate an array of cell fate decisions in the mammalian gonad. Thus, our results demonstrate that a previously unknown control mechanism of gene activity established an alternative system of sex determination.

### The recognition mechanism evolved by changing binding at two protein domains

Sex determination systems can evolve rapidly in vertebrates and invertebrates (*28*–*30*, *59*–*63*, *65*–*71*). One major question regarding this divergence of sex determination systems is how new sex determination genes evolutionarily gained their novel function, which has not been sufficiently resolved. Understanding the function of *csd* is a rare opportunity to reconstruct the molecular changes and evolutionary mechanism that led to a new sex determination mechanism. The *csd* gene originated from a gene duplication event from an ancestral copy of the *fem* gene (*21*, *22*, *46*). Gene duplication is a common mechanism underlying the origin of new sex determiner genes in invertebrates and vertebrates (*22*, *62*, *64*, *68*). We now found that two evolutionary novelties in the Csd protein, the CC domain and the polymorphic PSD, formed the new regulatory function for sex determination (Fig. 8B). In previous work, we showed by evolutionary nucleotide sequence analysis that six amino acid replacements gave evolutionary rise to the three heptad repeats, and this was shortly after the *csd* gene originated from gene duplication (*22*, *46*). We now found that these newly evolved repeats formed a CC motif among alleles, which is the shared binding element for the molecular recognition process. Furthermore, other evolutionary sequence analysis of *csd* alleles revealed that, on average, five to six adaptive amino acid differences in the RS domain together with HVR domain length differences were forming separate allele specificities (*18*). We now found that these allele-forming amino acid changes generate a distinct binding variant, which can only bind to itself but not to other variants. This nonbinding feature specifies the selective CC domain binding and recognition of different Csd protein variants. Collectively, we observe in this example that gain and losses of binding domains are evolutionary mechanisms underlying the origin of a new sex determination system.

## MATERIALS AND METHODS

### Honeybees and insect cells

The honeybees were from feral colonies of *A. mellifera* (western honeybee). Multiple *csd* alleles are present in these colonies, if not otherwise indicated. The colonies were located in the bee yard or the containment at the Heinrich-Heine University Düsseldorf. The transgenic queens were maintained together with worker bees in small mating nucleus hives (Segeberger nucs), which we kept in a secure containment (flight cage) so that transgenic animals were not able to escape into nature (S1 containment condition for the genetically modified organism (GMO) work). We applied the 3R principles (replacement, reduction, and refinement) to reduce the number of bees needed in our experiments. We hold the required approval, according to the law and relevant decrees. The *Sf*21 cells were maintained adherent in serum-free insect medium (Spodopan) supplemented with gentamycin (10 μg/ml).

### Transformation experimental procedure

Injection of piggyBac-derived transposon, handling of embryos, and rearing of queens followed our previously published procedures (*39*, *42*). We used between 15 and 30 pg of vector DNA for embryonic injection. *Csd* coding sequences were inserted into Bac[Am-actin5c] vector (*39*) via Not I/Avr II restriction sites. Queens carrying a different transgenic allele were generated (*39*, *42*) from eggs, which were randomly sampled in our bee yard. The transgenic queens were treated with CO_2_ for 7 min on two successive days. They were not inseminated. The CO_2_ treatment triggered the onset of egg laying. These queens produced only unfertilized eggs, which are haploids and carry only a single endogenous *csd* allele. The few transgenic genetic male progenies that carry the other, different *csd* allele coding sequence via the transgene (<10% of progeny carried the transgene) were identified by polymerase chain reactions (PCRs) and sequencing. The different allele combinations in haploid genetic males were characterized by PCR amplicons and sequencing (the *csd ^i,tg actin5C csd j^* males). To generate transgenic males in the F2 generation with identical *csd* coding sequence combinations, we started with a parental generation (P), which consisted of queens that were artificially inseminated with 10 drones, which all derived from a single mother queen (*18*). The *csd* coding sequences of these males were identified via reverse transcription PCRs (RT-PCRs). The amplicon was cloned and sequenced. These coding sequences (*csd* alleles 295-1 and 295-2) were used to test identical allele combinations in transgenic genetic males. To do so, F1 queens were produced from the parental generation (P) that carried transgenic either *csd* 295-1 or 295-2 sequences. Eight-day-old transgenic F1 queens were treated with CO_2_ for 7 min on two successive days to trigger the onset of egg laying. These queens laid only unfertilized eggs, which are haploid and are genetic males with a single endogenous *csd* allele. Transgenic genetic males of the F2 generation had a 50% chance to carry the identical *csd* allele than the endogenous one, which was determined by genotyping and sequencing (the *csd ^i,tg actin5C csd i^* genetic males). *csd* allele B2-25 was tested in male progenies from three queens, allele G3 in male progenies from two queens, allele 295-2 in male progenies from one queen, allele 295-1 in male progenies from one queen, allele B2-25 DQVEHR in male progenies from two queens, and allele G3 DQVEHR in male progenies from two queens.

### CRISPR-Cas9 experimental procedure

Cas9 protein (400 to 2000 ng/μl) was mixed with sgRNA2 at a molar ratio of 1:2 to 1:0.75 for injection (*38*). The sgRNA2 targets the *csd* gene at exon 2 and nucleotide position 325 of the coding sequence. sgRNA2 is predicted to have no off target (*38*), while mutations were independently induced in females. Single guide RNAs (sgRNAs) were generated as described (*38*) using the RiboMAX Kit and following the manufacturer’s instructions. RNAs were purified using the MEGAclear Kit. Larvae were in vitro reared under laboratory conditions at 34°C and 90% humidity using worker diet (*38*, *73*).

### DNA preparation, RNA isolation, cDNA synthesis, and PCR procedures

DNA was extracted using the peqGOLD Tissue DNA Mini Kit. RNA was isolated using TRIzol reagent. First strand cDNA synthesis was performed with RevertAid First Strand cDNA Synthesis Kit. If required, second-strand cDNA was synthesized using polymerase I and ribonuclease H. Double-stranded cDNA was purified using the EZNA Cycle Pure kit. Semiquantitative PCR amplifications were run under nonsaturating conditions and in technical triplicates for each bee sample.

### Sequencing and genotyping procedures

The sequences of cloned fragments were verified by repeated, double-strand sequencing of different clones using the Sanger method. Bees with a *csd* WT/stop genotypes were identified in a two-step process. Frameshift mutations were preselected by length polymorphism of hexachlorofluorescein-labeled amplicons, which were resolved on ABI 3130XL Genetic Analyzer. Length (base pair) analysis was performed using Peak Scanner software. Bees with a possible frameshift were deep-sequenced. At least 15,000 paired-end reads were generated for each amplicon and bee using the Illumina MiSeq machine. Unrelated sequences (less than 5% of the reads) were removed (*38*), and genotypes were determined from preprocessed data using IGV software.

### Coding sequence cloning procedures

*csd* coding sequences and the various fragments were cloned into pGBKT7 vector together with an myc-tag coding sequence via Eco RI/Sa lI restriction sites. If the sizes of expressed proteins were smaller than 12 kDa or size differences were too small to be resolved, then tags of 8-, 11-, or 15-kDa sizes were fused. These tags derived from a gentamicin-acetyltransferase protein (UniProtKB: P23181.1; 8 kDa, amino acids 1 to 76; 11 kDa, 1 to 103; and 15 kDa, 1 to 138), which showed no trimeric binding. To change the amino acids in the CC motif, mutations were introduced into oligonucleotide primer sequences before amplifications. Seamless ligation using Type IIS restriction enzymes (*74*) were used to combine the coding sequence from two PCR products. To generate YFP and Cer (cerulean fluorescent protein) fusion proteins, the coding sequences were inserted at the 3′ end of *csd* coding sequence using Eco RI/Not I and Xba I/Sac II restriction sites and the PIZV5-His vector. A spacer coding sequence (SP: GCGGCCGCGAGCTCACTAGTCATATGTTCTAGA) was inserted between *csd* and fluorescent protein coding sequences using Not I and Xba I restriction sites. Two *csd* expression cassettes were introduced into a single pFastBac HTA vector to ensure expressions of two Csd proteins from the same vector in a cell. Recombinations into baculovirus bacmid were performed following the manufacturer’s instruction.

### Oligomer-forming Western blot experimental procedure

Proteins were expressed from 300 to 600 ng of plasmid using 12.5 μl of T7 transcription/translation coupled reticulocyte lysate system. Protein extracts were prepared in native loading buffer (0.1 M tris, 10% glycerin, and 0.005% bromophenol blue) with no SDS. The native extracts were loaded and resolved using SDS-PAGE with oligomer-forming conditions (*45*), which we found using very low SDS concentration in the gel (0.01% SDS) and 0.15% SDS as running buffer. Proteins were transferred to polyvinylidene difluoride membrane. Blots were probed with primary mouse monoclonal anti-myc antibody, which was followed by goat anti-mouse secondary antibody with conjugated horseradish peroxidase. Immune complexes were visualized using chemiluminescence (ECL kit). Chemiluminescence was detected using LAS4000Mini (Fuji). For each lane, the chemiluminescence was measured, and the quantity of the Csd protein complexes and monomers were determined using Multi Gauche software.

### Fluorescence lifetime imaging microscopy and lifetime analysis

Baculovirus bacmid DNA and ROTI-Insectofect was added to 2 × 10^5^ *Sf*21 cells using Lab-Tek Chamber Slides. After 48 hours, cerulean fluorescence lifetime (τ) was measured (*43*). Fluorescence lifetime imaging microscopy was performed on a confocal laser scanning microscope (Olympus Fluoview1000) additionally equipped with a single-photon counting device with picosecond time resolution (PicoQuant Hydra Harp 400) (*75*). We followed procedures described in (*43*, *75*). We used a mono-exponential model function with two variables [fluorescence lifetime τ and scatter contribution *g* (*76*)], which we fitted using maximum likelihood estimator (MLE). We measured the instrument response function using the dye erythrosine. With this approach, we measured fluorescence lifetimes even under high background and low expression level conditions, as it is the case for Csd protein.

### Peptide detection procedure

Tissue lysis and sample preparation for mass spectrometry were performed as described elsewhere (*77*). Peptides were extracted with 1:2 acetonitrile/0.1% trifluoroacetic acid. Heavy isotope–labeled synthetic peptide (100 pmol; QYDQLHNVEEK) was added to each sample as internal reference.

Peptides were separated using a 120-min LC gradient with 300 nl/min (Ultimate 3000 Rapid Separation liquid chromatography system equipped with an Acclaim PepMap 100 C18 column). MS analysis was carried out on a Q-Exactive Plus mass spectrometer. Survey scans were carried out over a mass range from 150 to 2000 mass/charge ratio (*m*/*z*) at a resolution of 70,000 (at 200 *m*/*z*). After each full scan up to 5 parallel reaction monitoring (PRM) scans, targeting doubly and triply charged masses of the heavy and light variants of the target peptide, were performed at a resolution of 17,500 with a maximum ion time of 100 ms. Acquired spectra were searched using Mascot 2.4 software against the Swiss-Prot database, which included the sequence of the mutated allele.

### Quantification and statistical analysis

Images were analyzed using Multi Gauche software. All data are expressed as means and SEMs. Statistical comparisons were performed using Systat software. Fluorescence lifetime was analyzed using the software tools “AnI” and “Margarita” (software packages for multiparameter fluorescence spectroscopy and full correlation and multiparameter fluorescence imaging).
