# Supplementary material for: Recognition of polymorphic Csd proteins determines sex in the honeybee
Source: Sci Adv. 2023 Oct 4;9(40):eadg4239. doi: 10.1126/sciadv.adg4239 (PMC10550236; doi:10.1126/sciadv.adg4239)
Supplement: Supplementary file 1 — Tables S1 to S3 Figs. S1 to S12 [file sciadv.adg4239_sm.pdf]

Science Advances
AAAS

# Supplementary Materials for

## Recognition of polymorphic Csd proteins determines sex in the honeybee

Marianne Otte *et al.*

Corresponding author: Martin Beye, martin.beye@uni-duesseldorf.de

*Sci. Adv.* **9**, eadg4239 (2023)
DOI: 10.1126/sciadv.adg4239

**This PDF file includes:**

Tables S1 to S3
Figs. S1 to S12

# Supplementary Materials

## Table S1- S3

**Table S1. The number of *csd*$^{i/j\ stop}$ genetic females entering pupal stage.**

| | # at larval stage one | # at pupal stage |
|---|---|---|
| *csd*$^{i/j\ stop}$ female | 36 | 1 |
| wildtype female | 36 | 11 |
| wildtype male | 36 | 10 |

Brackets to the right of the table mark two comparisons, each labelled **: *csd*$^{i/j\ stop}$ female vs. wildtype female, and *csd*$^{i/j\ stop}$ female vs. wildtype male.

** $P$ < 0.01; Chi-square test

**Table S2: Amino acid sequences of the protein fragments used in figure 5 shown for the Csd D1-16 variant**

| | |
|---|---|
| D1-16 N-term | MKRNISNYSHHDEKFKQLRNEDNKIDLRSRTKEERLQHRREVWLIQQEREREHERLMKKMILEYELRRIREIEKLGSERSKSRSPGSRDRSNTSNTSKTVILSDKLESSDDISLFRGPEGIQINATELQKIKLEIHRDLPGKSTTTTVEVKRDIINPEDVILIRRTGEGSKPIFEREEIKNVLTKINKIEEHDTVLVVNIKKSGNESKKYATSSNSLRSRTHGFQHTS |
| D1-16 C-term | SRYSRERSCSRDRNREYKEKDRRYEKLHNEKEKLLEERTSRKRYSRSREREQNSYKNEREYRKYRERSKERSRDRTERERSREPKIISSLSNKTIHNNNNYNNNNYNNNYNNYNNNNYNNYKKLYYNIINIEQIPVPVPVPIYCGNFPPRPMGPWISIQEQIPRFRYIGPPTPFPRFIPPNAYRFRPPLNPRFGPTHQ |
| D1-16 RS | SRYSRERSCSRDRNREYKEKDRRYEKLHNEKEKLLEERTSRKRYSRSREREQNSYKNEREYRKYRERSKERSRDRTERERSREP |
| D1-16 ΔCCRS | LEERTSRKRYSRSREREQNSYKNEREYRKYRERSKERSRDRTERERSREP |
| D1-16 CC | RDRNREYKEKDRRYEKLHNEKEKLLEERT |
| D1-16 RSHV | SRYSRERSCSRDRNREYKEKDRRYEKLHNEKEKLLEERTSRKRYSRSREREQNSYKNEREYRKYRERSKERSRDRTERERSREPKIISSLSNKTIHNNNNYNNNNYNNNYNNYNNNNYNNYKKLYYNIINIEQIPVPVPVPIYCGNFPPRPMG |
| D1-16 HV | KIISSLSNKTIHNNNNYNNNNYNNNYNNYNNNNYNNYKKLYYNIINIEQIPVPVPVPIYCGNFPPRPMG |
| D1-16 HVPR | RDRTERERSREPKIISSLSNKTIHNNNNYNNNNYNNNYNNYNNNNYNNYKKLYYNIINIEQIPVPVPVPIYCGNFPPRPMGPWISIQEQIPRFRYIGPPTPFPRFIPPNAYRFRPPLNPRFGPTHQ |
| D1-16 PR | PWISIQEQIPRFRYIGPPTPFPRFIPPNAYRFRPPLNPRFGPTHQ |
| D1-16 ΔCC C-term | LEERTSRKRYSRSREREQNSYKNEREYRKYRERSKERSRDRTERERSREPKIISSLSNKTIHNNNNYNNNNYNNNYNNYNNNNYNNYKKLYYNIINIEQIPVPVPVPIYCGNFPPRPMGPWISIQEQIPRFRYIGPPTPFPRFIPPNAYRFRPPLNPRFGPTHQ |
| D1-16 PSD | ERSCSRDRNREYKEKDRRYEKLHNEKEKLLEERTSRKRYSRSREREQNSYKNEREYRKYRERSKERSRDRTERERSREPKIISSLSNKTIHNNNNYNNNNYNNNYNNYNNNNYNNYKKLYYNIINIEQIPVPVPVPIYCG |
| D1-16 Fr3-PSD | RIREIEKLGSERSKSRSPGSRDRSNTSNTSKTVILSDKLESSDDISLFRGPEGIQINATELQKIKLEIHRDLPGKSTTTTVEVKRDIINPEDVILIRRTGEGSKPIFEREEIKNVLTKINKIEEHDTVLVVNIKKSGNESKKYATSSNSLRSRTHGFQHTSSRYSRERSCSRDRNREYKEKDRRYEKLHNEKEKLLEERTSRKRYSRSREREQNSYKNEREYRKYRERSKERSRDRTERERSREPKIISSLSNKTIHNNNNYNNNNYNNNYNNYNNNNYNNYKKLYYNIINIEQIPVPVPVPIYCG |
| D1-16 Fr1-PSD | GEGSKPIFEREEIKNVLTKINKIEEHDTVLVVNIKKSGNESKKYATSSNSLRSRTHGFQHTSSRYSRERSCSRDRNREYKEKDRRYEKLHNEKEKLLEERTSRKRYSRSREREQNSYKNEREYRKYRERSKERSRDRTERERSREPKIISSLSNKTIHNNNNYNNNNYNNNNYNNNYNNYNNNNYNNYKKLYYNIINIEQIPVPVPVPIYCG |
| D1-16 N-term CC | MKRNISNYSHHDEKFKQLRNEDNKIDLRSRTKEERLQHRREVWLIQQEREREHERLMKKMILEYELRRIREIEKLGSERSKSRSPGSRDRSNTSNTSKTVILSDKLESSDDISLFRGPEGIQINATELQKIKLEIHRDLPGKSTTTTVEVKRDIINPEDVILIRRTGEGSKPIFEREEIKNVLTKINKIEEHDTVLVVNIKKSGNESKKYATSSNSLRSRTHGFQHTSSRYSRERSCSRDRNREYKEKDRRYEKLHNEKEKL |
| D1-16 N-term RS | MKRNISNYSHHDEKFKQLRNEDNKIDLRSRTKEERLQHRREVWLIQQEREREHERLMKKMILEYELRRIREIEKLGSERSKSRSPGSRDRSNTSNTSKTVILSDKLESSDDISLFRGPEGIQINATELQKIKLEIHRDLPGKSTTTTVEVKRDIINPEDVILIRRTGEGSKPIFEREEIKNVLTKINKIEEHDTVLVVNIKKSGNESKKYATSSNSLRSRTHGFQHTSSRYSRERSCSRDRNREYKEKDRRYEKLHNEKEKLLEERTSRKRYSRSREREQNSYKNEREYRKYRERSKERSRDRTERERSREP |
| D1-16 N-term PSD | MKRNISNYSHHDEKFKQLRNEDNKIDLRSRTKEERLQHRREVWLIQQEREREHERLMKKMILEYELRRIREIEKLGSERSKSRSPGSRDRSNTSNTSKTVILSDKLESSDDISLFRGPEGIQINATELQKIKLEIHRDLPGKSTTTTVEVKRDIINPEDVILIRRTGEGSKPIFEREEIKNVLTKINKIEEHDTVLVVNIKKSGNESKKYATSSNSLRSRTHGFQHTSSRYSRERSCSRDRNREYKEKDRRYEKLHNEKEKLLEERTSRKRYSRSREREQNSYKNEREYRKYRERSKERSRDRTERERSREPKIISSLSNKTIHNNNNYNNNNYNNNYNNYNNNNYNNYKKLYYNIINIEQIPVPVPVPIYCG |

**Table S3. The bindings of two different and two identical PSD variants.**

| Alleles | 295-1 | 295-2 | R3 | R1 | TPC1 | 701-2 |
|---|---|---|---|---|---|---|
| 295-1 | yes | n.d. | none | none | none | none |
| 295-2 | | yes | none | none | none | none |
| R3 | | | yes | n.d. | n.d. | none |
| R1 | | | | yes | n.d. | none |
| TPC1 | | | | | yes | n.d. |
| 701-2 | | | | | | yes |

# Figure S1 – S12

| Individual | Sequences at the target site of exon 2 |
| --- | --- |
| #I21 | reference TTATTTAGAGGACCAGAAGGTATTCAAATTAATGCAACAGAACTACA<br><br>csd i ...............A...............................<br>csd j stop ..................-............................ |
| #I23 | reference TTATTTAGAGGACCAGAAGGTATTCAAATTAATGCAACAGAACTACA<br><br>csd i ............TT.A...............................<br>csd j stop .........A........-............................ |
| #I24 | reference TTATTTAGAGGACCAGAAG-------------------GTATTCAAATTAATGC<br><br>csd i ...............A...-------------------................<br>csd j stop ...................TAAGTAGAAGTAAGTAGAA................<br>csd j ..................A-------------------................ |
| #I25 | reference TTATTTAGAGGACCAGAAGGTATTCAAATTAATGCAACAGAACTACA<br><br>csd i ...............A...............................<br>csd j stop .................-............................. |
| #I26 | reference TTATTTAGAGGACCAGAAGGTATTCAAATTAATGCAACAGAACTACA<br><br>csd i ...............A...............................<br>csd j stop ..................-............................<br>csd j ..................A............................ |
| #I33 | reference TTATTTAGAGGACCAGAAGGTATTCAAATTAATGCAACAGAACTACA<br><br>csd i ...............A...............................<br>csd j stop ..................-............................<br>csd j ..................A............................ |
| #I39 | reference TTATTTAGAGGACCAGAAGGTATTCAAATTAATGCAACAGAACTACA<br><br>csd i ............TT.A...............................<br>csd j stop ..................-............................ |
| #I50 | reference TTATTTAGAGGACCAGAAGGTATTCAAATTAATGCAACAGAACTACA<br><br>csd i ...............A...............................<br>csd j stop ...............----............................ |
| #I52 | reference TTATTTAGAGGACCAGAA--------GGTATTCAAATTAATGCAACAGAACTAC<br><br>csd i ...............A..--------............................<br>csd j stop ..................GAGAAATA............................<br>csd j ..................--------A........................... |
| #I65 | reference TTATTTAGAGGACCAGAAGGTATTCAAATTAATGCAACAGAACTACA<br><br>csd i ...............A...............................<br>csd j stop .................----.......................... |

**Figure S1. The nucleotide sequences of exon 2 of *csd*$^{i/j\ stop}$ genetic female mutants.** The target sequence of the sgRNAs is shown in the reference sequence (underlined sequence).

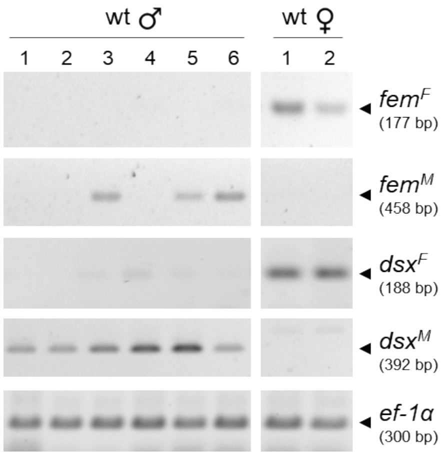


**Figure S2. The *fem$^M$* transcripts are not consistently detected in wt genetic males.**

A

| Allele tested | queen # | Sequence alignment |
|---|---|---|
| B2- 25 | 1 | tg B2-25 AGACAATATGAAAAATTACACAATGAAAAAGAAAAACTTTTAG<br>allele 1 ...........................................<br>allele 2 ....G...............T......................<br><br>AAGAAAGAACAAGTCGTAAGCGTTATTCTCGTTCAAGAGAACGAGAGCAAAAA<br>..........G..........................................<br>....................................................C<br><br>TCGTATAAAAATGAAAATTCTTATCGAAAGTATCGAGAAACATCGAAAGAACG<br>..A...........G..............A..............A........<br>.............................A.......................<br><br>ATCTCGAGATAGAACGGAACGAGAGAGATCTAGAGAACCTAAAATAATTTCAT<br>...............A................A-...................<br>...............A.....................................<br><br>CTTTATTGAACAAT-ACAATACATAATAATAATAATTATAAAAAATTAC----<br>......C.......T.....-T...................------TAATTA<br>......C......G-..........................------TAATAA<br><br>-----------------------AATATTACAATATTAATTATATTGAACAAA<br>TAATAATAATTATAATAAAAAATT.............................<br>TAATTATAATAATTATAAAAAATT......T.......T.A............<br><br>TTCCTGTTCCCGTTCCTATTCCTATTTATTGTGGGAATTTTCCACCAAGACCA<br>..........T......------..............................<br>..........T......G...................................<br><br>ATGGGACCTT<br>..........<br>.......... |
| | 2 | tg B2-25 AGACAATATGAAAAATTACACAATGAAAAAGAAAAACTTTTAG<br>allele 1 ....G...............................T......<br>allele 2 ....G...............T........G.............<br><br>AAGAAAGAACAAGTCGTAAGCGTTATTCTCGTTCAAGAGAACGAGAGCAAAAA<br>...............A......C.......A......................<br>...G......G............A.....................T......C<br><br>TCGTATAAAAATGAAAATTCTTATCGAAAGTATCGAGAAACATCGAAAGAACG<br>..A...........G..............A.......................<br>................GAGAA........A.......................<br><br>ATCTCGAGATAGAACGGAACGAGAGAGATCTAGAGAACCTAAAATAATTTCAT<br>...............A.....................................<br>................................A....................<br><br>CTTTATTGAACAAT-ACAATACATAATAATAA---------TAAT-----TAT<br>......C......G-.................---------....T---A..A<br>......C.......T.....-T...TC...T.CAATAATTA....AATTA..A<br><br>AAAAAATTAC-------------------------------------AATATT<br>T..T....CT------------AA--ATTAAAATATTAATTATATTG......<br>T..T.....TAATAATTATAATAATAATTAAAATATTAATTATATTG......<br><br>ACAATATTAATTATATTGAACAAATTCCTGTTCCCGTTCCTATTCCTATTTAT |

| | | |
|---|---|---|
| | | ..----------------AAAATT..........T......------......<br>..ATTATAATAATTATAAAAAATTAT...A....T......G...........<br><br>TGTGGGAATTTTCCACCAAGACCAATGGGACCTT<br>..................................<br>.................................. |
| | 3 | tg B2-25   AGACAATATGAAAAATTACACAATGAAAAAGAAAAACTTTTAG<br>allele 1   ....G...............T......................<br>allele 2   ....G...............T......................<br><br>AAGAAAGAACAAGTCGTAAGCGTTATTCTCGTTCAAGAGAACGAGAGCAAAAA<br>..........G..........................................<br>.....................................................<br><br>TCGTATAAAAATGAAAATTCTTATCGAAAGTATCGAGAAACATCGAAAGAACG<br>..A...........G..............A..............A........<br>.............................A.......................<br><br>ATCTCGAGATAGAACGGAACGAGAGAGATCTAGAGAACCTAAAATAATTTCAT<br>...............A................A....................<br>................................A....................<br><br>CTTTATTGAACAAT-ACAATACATAATAATAA------TAAT-----TATAAA<br>......C.......T.....-T..........TAATTA....AATTA..AT..<br>......C......G-.................------....---AA.TAT..<br><br>AAATTAC----------AATATTACAATATTAATTATATTGAACAAATTCCTG<br>T.....TAATAAAAAATT...................................<br>T.....TAATAAAAAATT..............T.A..................<br><br>TTCCCGTTCCTATTCCTATTTATTGTGGGAATTTTCCACCAAGACCAATGGGA<br><br>....T......------....................................<br>....T......G.........................................<br><br>CCTT<br>....<br>.... |
| G3 | 1 | tg G3      AGACGATATGAAAAATTACACAATGAAAAAGAAAAACTTTTAG<br>allele 1   ....................T......................<br>allele 2   ....................T...............T......<br><br>AAGAAAGAACGAGTTGTAAGCGTTATTCTCGTTCAAGAGAACGAGAGCAAAAG<br>...G..........C........A.............................<br>..........A...CA......C.......A...G..................<br><br>TTATATAAAAATGAAAGAGAATATCGAAAATATGGAGAAACATCGAAAGAACG<br>.CG..............................C.....G.............<br>.C............G.ATTCT............C...................<br><br>ATCTCGAAATAGAACAGAACGAGAAAAATCTAAAGAACCTAAAATAATTTCAT<br>.......G.......G........G.G..........................<br>.......G................G.G.....G....................<br><br>CTTTATCGAACAATTACAATT------------ACAACAATTATAATAATAAT<br>.....................ATAGCAATTATA.T..TT..A.....T.....<br>.............G-.....AC-----ATTATA.T..T...............<br><br>------------------------------------TATAAACCATTATATTA |

| | | |
|---|---|---|
| | | AATAACTATAATAATTATAATAATAATTATAATAAT......AA.........<br>------------------------------------.C....AA.........<br><br>CAATATTAATTATATTGAACAAATTCCTGTTCCTGTTCCTTTTCCTGTTTATT<br>............................A...........G.....A......<br>........................................------A......<br><br>ATGGGAATTTTCCACCAAGACCAATGGGACCTT<br>G................................<br>G................................ |
| | 2 | tg G3 AGACGATATGAAAAATTACACAATGAAAAAGAAAAACTTTTAG<br>allele 1 ......C.............................T......<br>allele 2 ...........................................<br><br>AAGAAAGAACGAGTTGTAAGCGTTATTCTCGTTCAAGAGAACGAGAGCAAAAG<br>..........A...C.......C.......A......................<br>.....GA...A...C.......C..............................<br><br>TTATATAAAAATGAAAGAGAATATCGAAAATATGGAGAAACATCGAAAGAACG<br>.C............G.ATTCT............C...................<br>.C............T.ATTCT............C......------------.<br><br>ATCTCGAAATAGAACAGAACGAGAAAAATCTAAAGAACCTAAAATAATTTCAT<br>.......G................G.G.....G....................<br>.......G................G.G...........G..............<br><br>CTTTATCGAACAATTACAATT------------ACAACAATTATAATAATAAT<br>.............G-.....AC-----ATAATA.T..T...............<br>..........T..........ATAGCAATAATT.T..T......T........<br><br>------------------TATAAACCATTATATTACAATATTAATTATATTGA<br>------------------.C....AA.......-...................<br>AATAATAATTATAATAAT......AA.................T.A.......<br><br>ACAAATTCCTGTTCCTGTTCCTTTTCCTGTTTATTATGGGAATTTTCCACCAA<br>......................------A......G.................<br>....C.................------.........................<br><br>GACCAATGGGACCTT<br>...............<br>............... |
| 295-1 | 1 | tg 295-1 AGACGATATGAAAAATTACACAATGAAAAAGAAAAACTTTTA<br>allele 2 ....................T...............T.....<br><br>GAAGAAAGAACGAGTCGTAAGCGCTATTCTCGTTCAAGAGAACGAGAGCGAAA<br>...........A....A..............A.................A...<br><br>ATCGTATAAAAATGAAAATTCTTATCGAAAATATCGAGAAACATCGAAAGAAC<br>...A...........G.....................................<br><br>GATCTCGAGATAGAACAGAACGAGAGAGATCTAGAGAACCTAAAATAATTTCA<br>.....................................................<br><br>TCTTTATCGAACAAGACAATACATAATAATAATAATTATAAATATAATTATAA<br>........................................-------------<br><br>TAATAATAATTATAATAATAATAATTATAATAATAATTATAATAATAATTGTA<br>------------------------------------------........C..<br><br>AAAAATTATATTACAATATTATTAATATTGAACAAATTCCTGTTCCTGTTCCT<br>.....................A.T............................. |

| | | |
|---|---|---|
| | | GTTCCTATTTATTGTGGGAATTTTCCACCAAGACCAATGGGACCTT<br>------........................................ |
| 295-2 | 1 | tg 295-2   AGACGATATGAAAAATTACACAATGAAAAAGAAAAACTTTTA<br>allele 2   ....................T.....................<br><br>GAAGGAAGAACGAGTCGTAAGCGTAATTCTCGTTCAAGAGAACGAGAGCAAAA<br>....A..................CT............................<br><br>CTCGTATAAAAATGAAAGAGAATATCGAAAATATCGAGAAACATCGAAAGAAC<br>A................ATTCT...............................<br><br>GATCTCGAGATAGAACAGAACGAGAGAGATCTAAAGAACCTAAAATAATTTCA<br>.................................G...................<br><br>TCTTTATCGAACAAT------TACAATTATAGCAATTATAA---TAATTATAA<br>..............GACAATAC.T...A...AT........ATA.........<br><br>TAATTATAATAATAATTATAATAATTATAATAATAATTATAATAATTA---TA<br>....A.....T.....A.............................A.TCG..<br><br>AAAAATTATATTACAATATTAATTATATTGAACAAATTCCTATTCCTGTTCCT<br>.....................T.A.................G...........<br><br>GTTCCTATTTATTGTGGGAATTTTCCACCAAGACCAATGGGACCTT<br>.............................................. |

B

| Allele tested | queen # | Sequence alignment |
|---|---|---|
| 295-1 | 1 | tg 295-2   AGACGATATGAAAAATTACACAATGAAAAAGAAAAACTTTTA<br>allele 1   ....................T.....................<br><br>GAAGAAAGAACGAGTCGTAAGCGCTATTCTCGTTCAAGAGAACGAGAGCGAAA<br>.................................................A...<br><br>ATCGTATAAAAATGAAAATTCTTATCGAAAATATCGAGAAACATCGAAAGAAC<br>.....................................................<br><br>GATCTCGAGATAGAACAGAACGAGAGAGATCTAGAGAACCTAAAATAATTTCA<br>.....................................................<br><br>TCTTTATCGAACAAGACAATACATAATAATAATAATTATAAATATAATTATAA<br>.....................................................<br><br>TAATAATAATTATAATAATAATAATTATAATAATAATTATAATAATAATTGTA<br>.....................................................<br><br>AAAAATTATATTACAATATTATTAATATTGAACAAATTCCTGTTCCTGTTCCT<br>.....................................................<br><br>GTTCCTATTTATTGTGGGAATTTTCCACCAAGACCAATGGGACCTT<br>.............................................. |
| 295-2 | 1 | tg 295-2   AGACGATATGAAAAATTACACAATGAAAAAGAAAAACTTTTA<br>allele 1   ..........................................<br><br>GAAGGAAGAACGAGTCGTAAGCGTAATTCTCGTTCAAGAGAACGAGAGCAAAA<br>.....................................................<br><br>CTCGTATAAAAATGAAAGAGAATATCGAAAATATCGAGAAACATCGAAAGAAC<br>..................................................... |

| | | |
|---|---|---|
| | | GATCTCGAGATAGAACAGAACGAGAGAGATCTAAAGAACCTAAAATAATTTCA<br>................G....................................<br><br>TCTTTATCGAACAATTACAATTATAGCAATTATAATAATTATAATAATTATAA<br>.....................................................<br><br>TAATAATTATAATAATTATAATAATAATTATAATAATTATAAAAAATTATATT<br>.....................................................<br><br>ACAATATTAATTATATTGAACAAATTCCTATTCCTGTTCCTGTTCCTATTTAT<br>.....................................................<br><br>TGTGGGAATTTTCCACCAAGACCAATGGGACCTT<br>.................................. |

**Figure S3. The combinations of nucleotide coding sequences that were tested in *csd* $^{i,\ tg\ actin5C\ csd\ j}$ (A) and *csd* $^{i,\ tg\ actin5C\ csd\ i}$ (B) genetic males.** The nucleotide sequences coding for PSD are presented. The nucleotide differences in combinations of identical alleles (*csd* $^{i,\ tg\ actin5C\ csd\ i}$ males) are silent differences.

```
B2-25      1 MKRNISNYSHHDEKFKQLRNEDNKIDLRSRTKEERLQHRREVWLIQQEREREHERLMKKM  60
D1-16      1 ............................................................  60
295-1      1 ......S...............SE.................A...........Q......  60
295-2      1 ......S..............NSE.................A...........Q......  60
G3         1 ......S...............SE.................A...........Q......  60
D2-27      1 ......S...............SE.................A...........Q......  60

B2-25     61 ILEYELRRIREIEKLGSERSKSRSPDSRDRSNTSNTSKTVILSNK-----------GPEG 109
D1-16     61 .........................G.................D.LESSDDISLFR.... 120
295-1     61 ................................A..........D.LESSDDISLFR.... 120
295-2     61 ......................T.........A..........D.LESSDDISLFR.... 120
G3        61 ................................A..........D.LESSDDISLFR.... 120
D2-27     61 ................................A..........D.LESSDDISLFR.... 120

B2-25    110 IQINATELQKIKLEIHRDLPGKSTTTTVEVKRDIINPEDVIVIRRTGEGSKPLFEREEIK 169
D1-16    121 .........................................L..........I....... 180
295-1    121 .........................................L..........I....... 180
295-2    121 .........................................L..........I....... 180
G3       121 .........................................L..........I....... 180
D2-27    121 ...........................A.....T.......L..........I....... 180

B2-25    170 NVLTKINKIEEHDTVLVVNIEKSGKESKKYATSSNSLRNRTHGFQHTSSRYSRERSCSRD 229
D1-16    181 ....................K...N.............S..................... 240
295-1    181 .........K..............N.............S...D................. 240
295-2    181 .........K..............N................................... 240
G3       181 ....................K...N.............S..........H.......... 240
D2-27    181 ....................K...N.............S..........H.......... 240

B2-25    230 RNREYRKKDRQYEKLHNEKEKLLEERTSRKRYSRSREREQKSYKNENSYRKYRETSKERS 289
D1-16    241 .....KE...R.............................N.....RE......R..... 300
295-1    241 .....KE...R............................R.................... 300
295-2    241 .....K....R.............G......N........N.....RE............ 300
G3       241 ..........R.................C............L....RE....G....... 300
D2-27    241 ..........R.................C............L....RE....G....... 300

B2-25    290 RDRTERERSREPKII-----------------SSL-LNNTIH------------------ 313
D1-16    301 ...............-----------------...-S.K...NNNNYNNNNYNNNYNNYN 342
295-1    301 ...............-----------------...-S.K...NNNNYKYNYNNNNYNNNN 342
295-2    301 .........K.....SSLSNNYNYSNYNNYNNYNN-NY.NYN------------------ 341
G3       301 .N.....K.K.....-----------------...SN.YNYN------------------ 325
D2-27    301 .N.....K.K.....-----------------...SN.YNYN------------------ 325

B2-25    314 ----NNNNYKKLQYYNINYIEQIPVPVPIPIYCGNFPPRPMGPWISIQEQIPRFRYIGPP 369
D1-16    343 NN--.Y......-....IN.........V............................... 399
295-1    343 YNNNY...C...-....IN.........V............................... 401
295-2    342 ----..Y.NY.KL...........I...V..........................H...S 397
G3       326 ----.Y..NY.PL...............F.V.Y..........FV............... 381
D2-27    326 ----.Y..NY.PL...............F.V.Y..........FV........L...... 381
```

```
B2-25        370 TPFPRFIPPNAYRFRPPLNPRFGPTYQ 396
D1-16        400 .........................H. 426
295-1        402 .........................H. 428
295-2        398 .........................H. 424
G3           382 .S..............S......S.H. 408
D2-27        382 .S..............S......S.H. 408
```

**Figure S4. The *csd* alleles used in the transgenic and protein studies.** The coding sequences were translated and aligned according to amino acid identities.

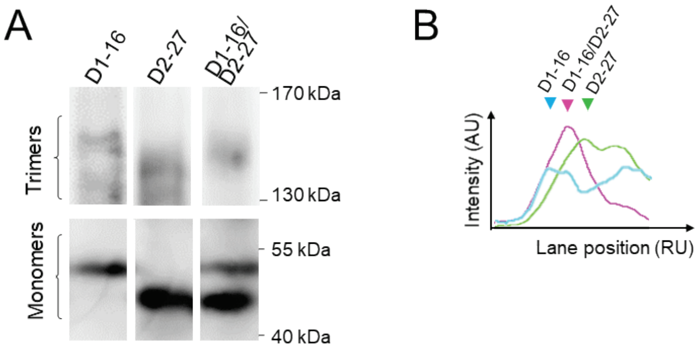


**Figure S5. Different Csd protein variants form complexes of intermediate size.**

(A) Highly resolved trimeric complexes of different and single Csd protein variant expressions in nonreducing Western blot using 6% acrylamide gels and anti-myc antibody staining. Lanes are from the same Western blot.

(B) Detected intensities along the length position of each lane (lane profile) and this for the western blot shown in (A). Arrows mark the peak of intensities for the different conditions. AU, arbitrary units. RU, relative units.

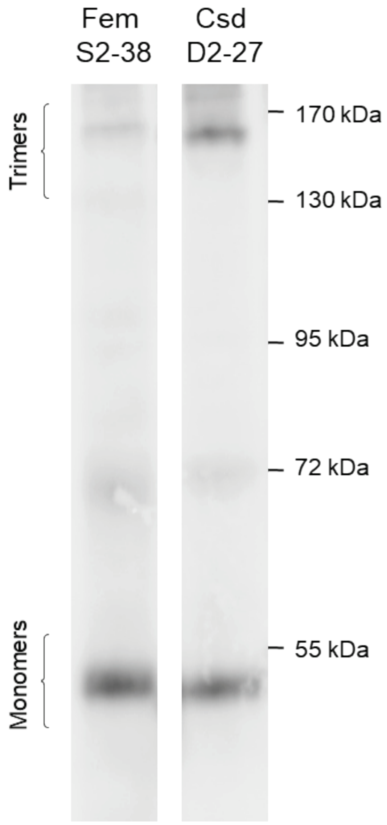


**Figure S6. The Fem proteins form low amounts of trimers.**

Nonreducing Western blots were stained with anti-myc antibody. Lanes are from the same Western blot.

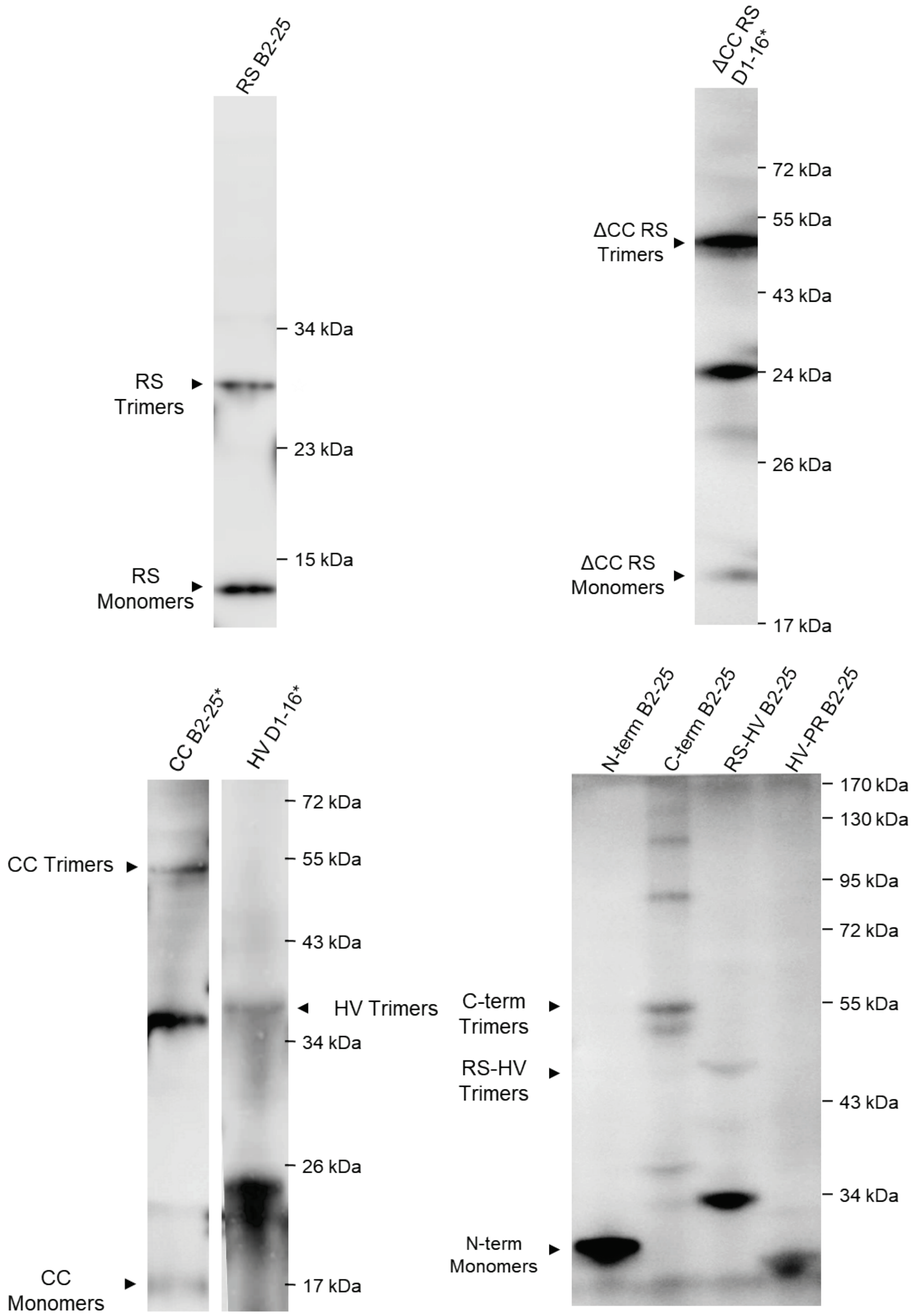


**Figure S7. Fragments of PSD have homotrimeric binding abilities**.
Nonreducing Western blots were stained with anti-myc antibody. Lanes from the same Western blot are shown in a single picture. The domains are schematically presented in Figure 4A. * marks domains that were further enlarged with tags.

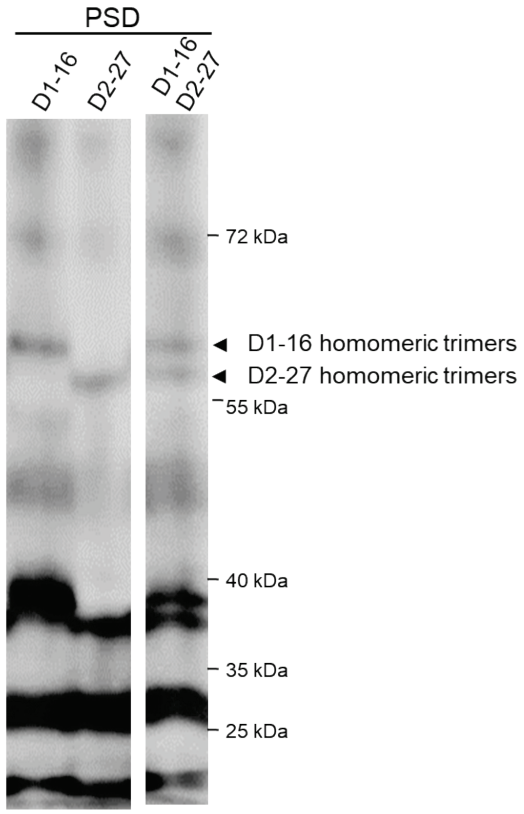


**Figure S8. The different PSD variants did not form heterotrimeric complexes.** Example of a nonreducing Western blot of PSD that were stained with anti-myc antibody. Lanes are from the same Western blot. Either single PSD variant and combination of two different PSDs were expressed. Trimers in two expressions were of homotrimeric and not intermediate, heteromeric size.

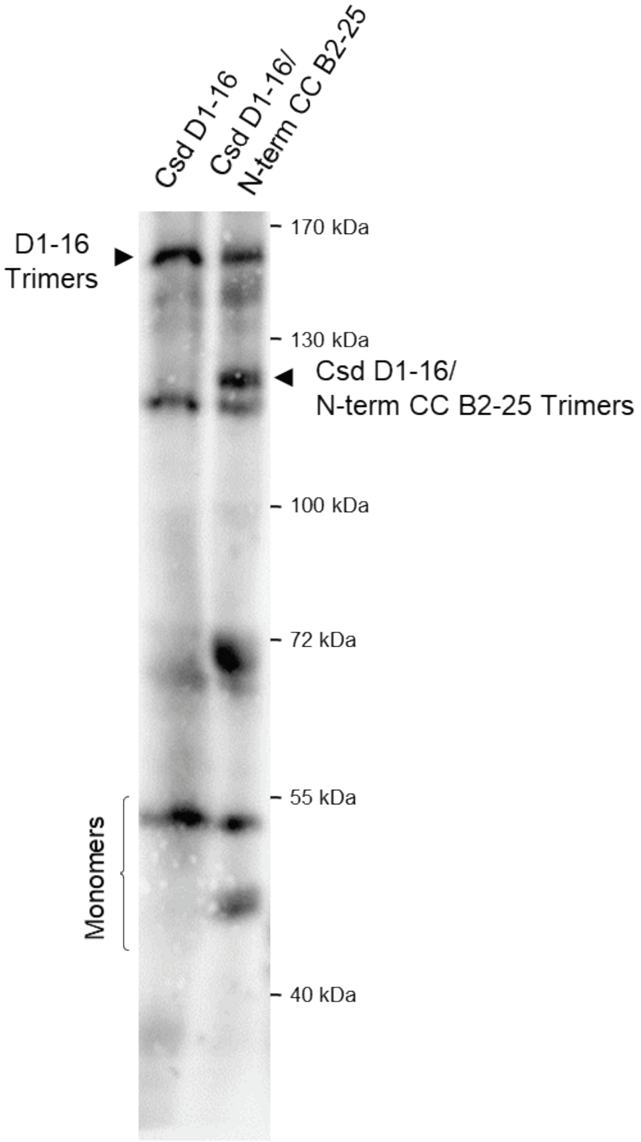


**Figure S9. The N-terminus/CC fragment forms together with full-length proteins heteromeric complexes.** A nonreducing Western blot using anti-myc antibody staining is shown.

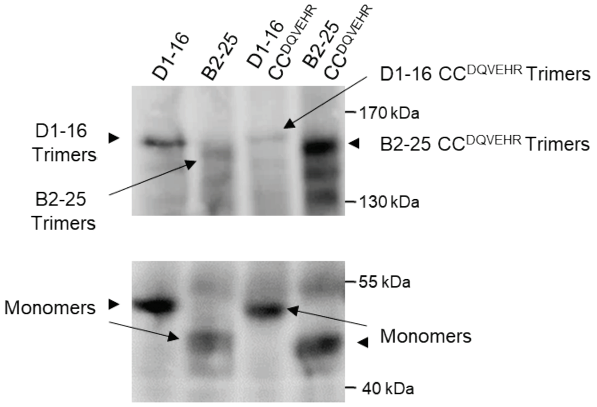


**Figure S10. Single Csd CC$^{DQVEHR}$ protein variants form homotrimeric complexes.** Nonreducing Western blot of single protein expressions using anti-myc antibody staining is shown.

| Allele tested | queen # | Sequence alignment |
|---|---|---|
| B2-25 $CC^{DQVEHR}$ | 1 | B2-25 $CC^{DQVEHR}$ AGACAATATGACCAATTACACAATGTAGAAGAAAAACAT<br>allele 1 ....G......AA.......T....A.A.........T.<br>allele 2 ....G......AA............A.A.........T.<br><br>TTACGAGAAAGAACAAGTCGTAAGCGTTATTCTCGTTCAAGAGAACGAGAGCA<br>...GA..G..................C..........................<br>...GA.........G......................................<br><br>AAAATCGTATAAAAATGAAAATTCTTATCGAAAGTATCGAGAAACATCGAAAG<br>...G..A...........G..............A................G..<br>......A...........G..............A..............A....<br><br>AACGATCTCGAGATAGAACGGAACGAGAGAGATCTAGAGAACCTAAAATAATT<br>..................AA....---.........A.....G..........<br>...................A................A................<br><br>TCATCTTTATTGAACAAT-ACAATACATAATAATAA------TAAT-----TA<br>..........C.......T...T.-TC...C.T.TC------....TATAA..<br>..........C.......T.....-T..........TAATTA....AATTA..<br><br>TAAAAAATTAC----------------------AATATTACAATATTAATTAT<br>AT..T....CT---------------AAAAAATT...................<br>AT..T.....TAATAATAATTATAATAAAAAATT...................<br><br>ATTGAACAAATTCCTGTTCCCGTTCCTATTCCTATTTATTGTGGGAATTTTCC<br>....................TA.....G.....G...................<br>....................T......------....................<br><br>ACCAAGACCAATGGGACCTT<br>....................<br>.................... |
| | 2 | B2-25 $CC^{DQVEHR}$ AGACAATATGACCAATTACACAATGTAGAAGAAAAACAT<br>allele 1 ....G......AA.......T....A.A........TT.<br>allele 2 ....G......AA............A.A.........T.<br><br>TTACGAGAAAGAACAAGTCGTAAGCGTTATTCTCGTTCAAGAGAACGAGAGCA<br>...GA..............A......C.......A..................<br>...GA...C.......................C....................<br><br>AAAATCGTATAAAAATGAAAATTCTTATCGAAAGTATCGAGAAACATCGAAAG<br>......A...........G..............A...................<br>...C................GAGAA........A...................<br><br>AACGATCTCGAGATAGAACGGAACGAGAGAGATCTAGAGAACCTAAAATAATT<br>...................A.................................<br>.....................................................<br><br>TCATCTTTATTGAACAAT-ACAATACATAATAATAATAAT-----TATAAAAA<br>..........C......G-.....................T---A..AT..T.<br>..........C...T...T.....-T...GC...T.....AATTA..AT..T.<br><br>ATTAC-------------------AATATTACAATATTAATTATATTGAACAA<br>...CT------------AAAAAATT............................<br>....TAATAATTATAATAAAAAATT............................<br><br>ATTCCTGTTCCCGTTCCTATTCCTATTTATTGTGGGAATTTTCCACCAAGACC<br>...........T......------G............................<br>...........T......G....CG............................ |

| | | |
|---|---|---|
| | | AATGGGACCTT<br>...........<br>........... |
| G3 CC$^{DQVEHR}$ | 1 | G3 CC$^{DQVEHR}$ AGACGATATGACCAATTACACAATGTAGAAGAAAAACATTTAC<br>allele 1   ...........AA.......T....A.A.......GTT....G<br>allele 2   ...........AA.......T....A.A.........T....G<br><br>GAGAAAGAACGAGTTGTAAGCGTTATTCTCGTTCAAGAGAACGAGAGCAAAAG<br>A.........A...C..G...................................<br>A.........A...C......................................<br><br>TTATATAAAAATGAAAGAGAATATCGAAAATATGGAGAAACATCGAAAGAACG<br>.C............G......................................<br>.CG..............................C...................<br><br>ATCTCGAAATAGAACAGAACGAGAAAAATCTAAAGAACCTAAAATAATTTCAT<br>..............G.........G.G.....G....................<br>G......G................G.G.....G....................<br><br>CTTTATCGAACAATTACAATT---------ACAACAATTATAATAATAAT---<br>.........G...........CTAACAATT.T..T.......G..C....---<br>.............G-.....AC--ATAATA.T..T..A......T.....AAA<br><br>------------------------------TATAAACCATTATATTACAATAT<br>------------------------TATAAAC.ATT..T..AT.G.........<br>TATAATTATAATAATAATAATTATAATAAT......AA...............<br><br>TAATTATATTGAACAAATTCCTGTTCCTGTTCCTTTTCCTGTTTATTATGGGA<br>......................A...........------A......G.....<br>......................A...........------A......G.....<br><br>ATTTTCCACCAAGACCAATGGGACCTT<br>...........................<br>............................ |
| | 2 | G3 CC$^{DQVEHR}$ AGACGATATGACCAATTACACAATGTAGAAGAAAAACATTTAC<br>allele 1   ....A......AA..C.........A.A.........T....G<br>allele 2   ...........AA.......T....A.A.........T....G<br><br>GAGAAAGAACGAGTTGTAAGCGTTATTCTCGTTCAAGAGAACGAGAGCAAAAG<br>A.........A...C......................................<br>A.............C..................................G...<br><br>TTATATAAAAATGAAAGAGAATATCGAAAATATGGAGAAACATCGAAAGAACG<br>.CG.............ATTCT............C...................<br>.CG..............................C...................<br><br>ATCTCGAAATAGAACAGAACGAGAAAAATCTAAAGAACCTAAAATAATTTCAT<br>.......G................G.G.....G....................<br>.......G.......G..........G..........................<br><br>CTTTATCGAACAATTACAATT-------ACAACAATTATAATAATAAT-----<br>.............G-.....ACATAATA.T..T...............AATTA<br>..........T...-.....ACATA---.T..T........AT.....-----<br><br>-------------------------------TATAAACCATTATATTACAATA<br>TAATAATTATAATAATAATAATTATAATAAT......AA..AT.G........<br>----------TATAATAATAATTATAATAAT.....TAA..............<br><br>TTAATTATATTGAACAAATTCCTGTTCCTGTTCCTTTTCCTGTTTATTATGGG<br>...................................G.....A......G....<br>......................C............G.....A......G.... |

| | | |
|---|---|---|
| | | AATTTTCCACCAAGACCAATGGGACCTT<br>............................<br>............................ |

**Figure S11. The combination of different *csd* nucleotide coding sequences with mutated CC domain that were tested in transgenic *csd* $^{i,\ tg\ actin5C\ csd\ j\ CC\ DQVEHR}$ genetic males.** The nucleotide sequences coding for PSDs are presented.

A

Csd B2-25 CC 237-K D R Q Y **E K** L H N **E K** E K **L** L **E** E-254

Csd B2-25 CC$^{DQVEHR}$ 237-K D R Q Y **D Q** L H N **V E** E K **H** L **R** E-254

B

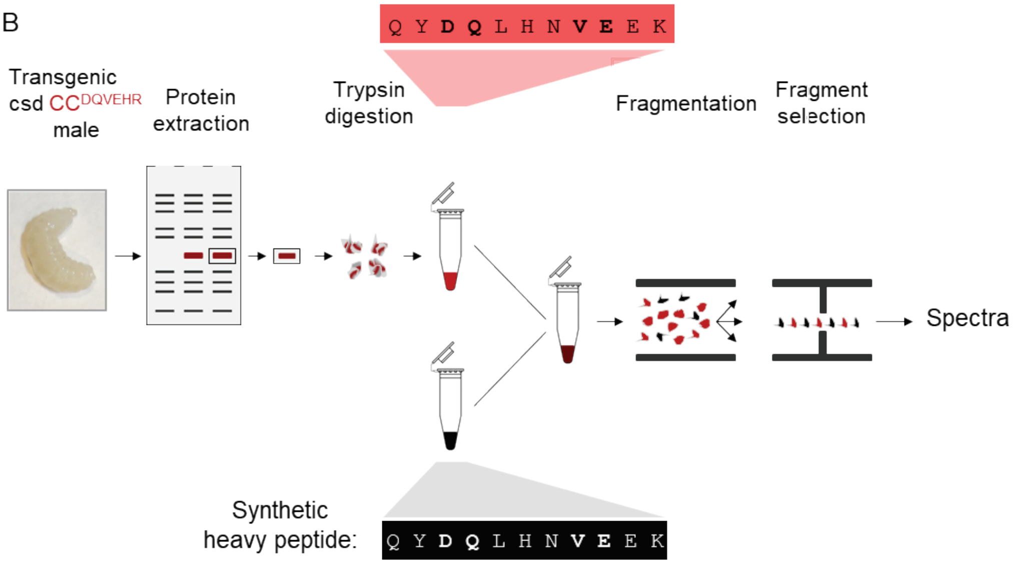


C CC$^{DQVEHR}$ peptide ion table

| # | b | b-H2O | b-NH3 | b (2+) | Seq | y | y-H2O | y-NH3 | y (2+) | # |
|---|---|---|---|---|---|---|---|---|---|---|
| 1 | 129.10 | 111.08 | 112.04 | 65.03 | Q | | | | | 11 |
| 2 | 292.13 | 274.12 | 275.10 | 146.56 | Y | 1274.60 | 1256.59 | 1257.57 | 637.80 | 10 |
| 3 | 407.16 | 389.15 | 390.13 | 204.08 | D | 1111.54 | 1093.53 | 1094.51 | 556.27 | 9 |
| 4 | 535.21 | 517.20 | 518.20 | 268.11 | Q | 996.51 | 978.50 | 979.53 | 498.76 | 8 |
| 5 | 648.30 | 630.29 | 631.28 | 324.65 | L | 868.48 | 850.44 | 851.43 | 434.23 | 7 |
| 6 | 785.35 | 767.35 | 768.33 | 393.18 | H | 755.37 | 737.36 | 738.34 | 378.18 | 6 |
| 7 | 899.40 | 881.39 | 882.38 | 450.20 | N | 618.31 | 600.30 | 601.28 | 309.65 | 5 |
| 8 | 998.52 | 980.50 | 981.51 | 500.18 | V | 504.27 | 486.26 | 487.24 | 253.09 | 4 |
| 9 | 1127.51 | 1109.50 | 1110.49 | 564.26 | E | 405.20 | 387.19 | 388.17 | 203.10 | 3 |
| 10 | 1256.55 | 1238.54 | 1239.53 | 628.78 | E | 276.16 | 258.14 | 259.09 | 138.58 | 2 |
| 11 | | | | | K | 147.11 | 129.10 | 130.05 | 74.06 | 1 |

D Spectrum heavy peptide

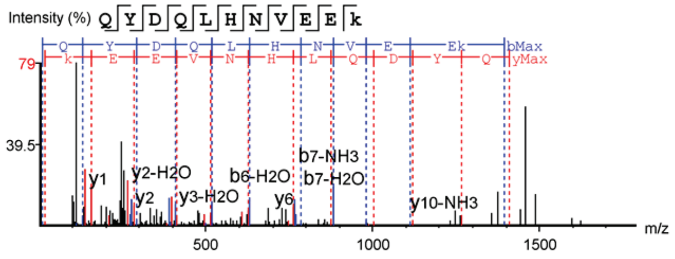


**Figure S12. The detection of the transgenetically expressed CC$^{DQVEHR}$ peptide in the bees using MALDI TOF spectra**.

(A) The sequence of the diagnostic peptide sequence.

(B) Workflow of the procedures. Isotope-labeled synthetic peptide of the same sequence was employed to compare spectra.

(C) Peptide ion table.

(D) Spectra heavy peptide.
